# Effects of glycemic traits on left ventricular structure and function: a mendelian randomization study

**DOI:** 10.1186/s12933-022-01540-6

**Published:** 2022-06-17

**Authors:** Sizhi Ai, Xiaoyu Wang, Shanshan Wang, Yilin Zhao, Shuxun Guo, Guohua Li, Zhigang Chen, Fei Lin, Sheng Guo, Yan Li, Jihui Zhang, Guoan Zhao

**Affiliations:** 1 Department of Cardiology, Life Science Research Center, The First Affiliated Hospital of Xinxiang Medical University, Weihui, China; 2 Center for Sleep and Circadian Medicine, The Affiliated Brain Hospital of Guangzhou Medical University, Guangzhou, Guangdong China; 3 Guangdong Mental Health Center, Guangdong Provincial People’s Hospital Guangdong Academy of Medical Sciences, Guangzhou, Guangdong China; 4 Li Chiu Kong Family Sleep assessment Unit, Department of Psychiatry, Faculty of Medicine, The Chinese University of Hong Kong, Shatin, Hong Kong SAR China

**Keywords:** Glycemic traits, Insulin resistance, Left ventricular structure and function, Mendelian randomization

## Abstract

**Background:**

Adverse ventricular structure and function is a key pathogenic mechanism of heart failure. Observational studies have shown that both insulin resistance (IR) and glycemic level are associated with adverse ventricular structure and function. However, whether IR and glycemic level are causally associated with cardiac structure and function remains unclear.

**Methods:**

Genetic variants for IR, fasting insulin, HbA1c, and fasting glucose were selected based on published genome-wide association studies, which included 188,577, 108,557, 123,665, and 133,010 individuals of European ancestry, respectively. Outcome datasets for left ventricular (LV) parameters were obtained from UK Biobank Cardiovascular Magnetic Resonance sub-study (n = 16,923). Mendelian randomization (MR) analyses with the inverse-variance weighted (IVW) method were used for the primary analyses, while weighted median, MR-Egger, and MR-PRESSO were used for sensitivity analyses. Multivariable MR analyses were also conducted to examine the independent effects of glycemic traits on LV parameters.

**Results:**

In the primary IVW MR analyses, per 1-standard deviation (SD) higher IR was significantly associated with lower LV end-diastolic volume (β = − 0.31 ml, 95% confidence interval [CI] − 0.48 to − 0.14 ml; *P* = 4.20 × 10^−4^), lower LV end-systolic volume (β = − 0.34 ml, 95% CI − 0.51 to − 0.16 ml; *P* = 1.43 × 10^−4^), and higher LV mass to end-diastolic volume ratio (β = 0.50 g/ml, 95% CI 0.32 to 0.67 g/ml; *P* = 6.24 × 10^−8^) after Bonferroni adjustment. However, no associations of HbA1c and fasting glucose were observed with any LV parameters. Results from sensitivity analyses were consistent with the main findings, but with a slightly attenuated estimate. Multivariable MR analyses provided further evidence for an independent effect of IR on the adverse changes in LV parameters after controlling for HbA1c.

**Conclusions:**

Our study suggests that genetic liability to IR rather than those of glycemic levels are associated with adverse changes in LV structure and function, which may strengthen our understanding of IR as a risk factor for heart failure by providing evidence of direct impact on cardiac morphology.

**Supplementary Information:**

The online version contains supplementary material available at 10.1186/s12933-022-01540-6.

## Background

Heart failure is a leading cause of mortality worldwide [1]. Despite the progress in diagnosis and treatment, the 5-year mortality rate of hospitalized heart failure patients is still more than 50%, which points to the importance of effective prevention [2]. Adverse left ventricular (LV) structure and function, such as LV hypertrophy which starts years to decades before the onset of heart failure symptoms, is the key pathogenic mechanism of heart failure [3]. Recent heart failure guidelines emphasize the need to better understand and manage risk factors triggering ventricular structure and function [4]. Observational studies have shown that glycemic traits related to type 2 diabetes, such as insulin resistance (IR), hemoglobin A1c (HbA1c), and fasting glucose, are strongly associated with adverse ventricular structure and function [5]. However, the observational study design is vulnerable to reverse causality and residual confounding effects [6]. Therefore, whether IR, HbA1c, and fasting glucose are causally associated with cardiac structure and function remains unclear.

Mendelian randomization (MR), using genetic variants as instrumental variables for exposure, can be used to provide less confounded results [7]. Genetic variation is determined by the random allocation of alleles during conception and unlikely to be affected by confounding factors. A recent MR study has shown that genetic liability to IR is associated with increased heart failure risk, whereas no significant associations have been found between HbA1c or fasting glucose with heart failure [8]. However, the pathophysiological pathway underlying the causal association between glycemic traits and heart failure is still unclear.

In view of the key pathogenic role of ventricular structure and function on heart failure [3], the present study aimed to examine the potential effects of genetic liability to glycemic traits (such as IR, fasting insulin, HbA1c, and fasting glucose) with LV structure and function, which may strengthen our understanding of glycemic traits as risk factors for heart failure.

## Methods

This two-sample MR study was conducted based on publicly available summary data from genome-wide association studies (GWASs) consortia [9–13]. Table 1 shows the detailed clinical information of these GWASs. The detailed methods of two-sample MR have been described in previous study [14]. Conducting MR analyses, the selected genetic variants need to follow three key assumptions: (1) relevance assumption: genetic variants are closely related to the exposure of interest; (2) independence assumption: genetic variants are not related to any confounding factors that affect the exposure-outcome association; and (3) exclusion restriction: genetic variants cause no effect on outcomes unless via their effect on the exposure of interest [15].

**Table 1:** Characteristics of the GWASs used in present study

Phenotype	PMID	Consortium	Sample size	Ancestry	Exclusion criteria	Adjusted covariates
Exposure						
IR	27841877	MAGIC for fasting insulin adjusting BMI	108,557	European	Individuals had a physician diagnosis of diabetes, were on diabetes treatment (oral or insulin), or had a fasting plasma glucose equal to or greater than 7 mmol/l	Age, sex, BMI
	29046328	GLGC for TGs and HDL-C	188,577	European	Individuals known to be on lipid-lowering medications	Age, sex
HbA1c	28898252	MAGIC	123,665	European	Individuals had a physician diagnosis of diabetes, were on diabetes treatment (oral or insulin), or had a fasting plasma glucose equal to or greater than 7 mmol/l	Study-specific covariates, age, sex, and genomic control
Fasting insulin	22885924	MAGIC	108,557	European	Individuals had a physician diagnosis of diabetes, were on diabetes treatment (oral or insulin), or had a fasting plasma glucose equal to or greater than 7 mmol/l	Age, sex, BMI
Fasting glucose			133,010			
Outcomes						
LV end-diastolic volume	31554410	UK Biobank	16,920	European	Individuals with prevalent myocardial infarction, heart failure, or LV ejection fraction < 50% to minimize the confounding influence of these preexisting conditions were excluded	Age, sex, height, weight, systolic blood pressure, phenotype-derivation method, array type, and imaging center
LV end-systolic volume			16,920			
LV ejection fraction			16,923			
LV mass			16,920			
LV mass to end-diastolic volume ratio			16,884			
Heart failure	31919418	HERMES	47,309 cases and 930,014 controls	European	Non-European ancestry individuals were excluded	Age, sex, genotyping array and the first 10 principal components

*GWAS* genome-wide association studies, *IR* insulin resistance, *HDL-C* high-density lipoprotein cholesterol, *TGs* triglycerides, *HbA1c* hemoglobin A1c, *LV* left ventricular, *BMI* body mass index, *MAGIC* the Meta-Analyses of Glucose and Insulin-related traits Consortium, *GLGC* Global Lipids Genetics Consortium, *HERMES* the Heart Failure Molecular Epidemiology for Therapeutic Targets

### Genetic instrument selection

IR is a complex trait that can be assessed using different indicators, such as the hyperinsulinemic-euglycemic clamp technique (the golden standard), insulin sensitivity test (based on oral glucose tolerance test), homeostatic model assessment of IR, and fasting insulin. Previous GWAS of the golden standard measure of IR (sample size = 5624) is limited by sample size and cannot provide sufficient power for MR estimate [16]. As a clinical condition of impaired insulin sensitivity, IR is not only characterized by compensatory increases in insulin but also frequently accompanied by dyslipidemia [9, 17]. A dyslipidemia pattern with higher triglycerides (TGs) levels and lower high-density lipoprotein cholesterol (HDL-C) levels is considered to be one of the significant clinical features of IR [17], and the TG to HDL-C ratio has also been proven to be a better predictor of IR [18]. In the GWAS of fasting insulin conducted by Scott et al.[12], they found that 10 of 19 single nucleotide polymorphisms (SNPs) associated with fasting insulin were also significantly associated with higher TG and lower HDL-C levels. A genetic instrument based on these 10 SNPs has also been demonstrated to associate with IR metrics measured by the golden standard [19]. Therefore, the combination of these three IR phenotypes (higher fasting insulin, higher TGs, and lower HDL-C) may be used to identify specific genetic determinants of IR [9, 19]. Lotta et al. [9] identified a total of 53 SNPs associated with these three components of IR phenotype (higher fasting insulin adjusted for body mass index [BMI], higher TGs and lower HDL-C at *P* < 0.005 for each trait) in up to 188,577 European individuals. Each of 53 SNPs was the lead insulin-associated SNP at each 1 Mb region, all of the SNPs were located in distinct genomic regions [9]. The 53 SNPs have been used in several studies to examine the causal effects of IR with other diseases [6, 10, 20, 21]. In the present study, we also used these 53 SNPs as instrumental variables for IR. Given that Lotta et al. [9] did not provide effect estimates nor corresponding standard errors for the associations of these SNPs with the IR phenotype, we obtained the effect of SNPs on exposure estimation from the study of Wang et al. [10]. To generate the estimate of each SNP association with IR phenotypes, Wang et al. [10] first obtained the beta-coefficient for each SNP associated with three components of IR phenotypes (i.e. fasting insulin adjusted for BMI from the MAGIC consortia, TGs and HDL-C from the GLGC consortia), and standardized the beta coefficient of each SNP association. Then, they meta-analyzed the absolute values of the standardized beta coefficient of each SNP associations with fasting insulin (adjusted for BMI), TGs and HDL-C via the fixed effect inverse-variance method, which served as the beta coefficient of IR.

Because IR is a composite phenotype in this study, we conducted several additional sensitivity analyses to address the potential violations of our assumption: (1) Most of the 53 SNPs contributed similarly to each trait of the composite IR phenotype except for rs1011685 (near *LPL*), which had a much weaker effect on insulin adjusted for BMI [10, 21]. Therefore, we conducted sensitivity analyses in which rs1011685 was excluded from the instrumental variable; (2) Considering the close association of obesity with IR [10], and the important role of obesity in cardiac remodeling [22], we further excluded 9 SNPs that individually associated with BMI at a threshold of *P* < 0.001 using GWAS summary data from GIANT consortia [9, 10, 23]; (3) Meta-GWAS allows the selection of SNPs based on pleiotropy, thus, SNPs can mark heterogeneous pathways [24], but some of them may lead to unbalanced horizontal pleiotropy. In the present study, 25 of the 53-SNPs have been previously reported to be associated with TGs or HDL-C at genome-wide significance level [25]. These lipid-associated SNPs may bias our inferences toward the associations between lipids and LV parameters. Therefore, we conducted additional analyses using the rest 28-SNPs as instrumental variable [10, 21]; (4) To further minimize the potential impact of horizontal pleiotropy of SNP on MR estimates, we used the PhenoScanner tool (http://www.phenoscanner.medschl.cam.ac.uk/) [26] to check whether any of the 53 SNPs was associated with other potential confounders affecting ventricular structure and function. We assessed SNPs at a threshold of *P* < 5 × 10^−8^ for their associations with other potential confounders, such as obesity [22], hypertension [27], and coronary artery disease [28]. Additional file 1: Table S1 shows SNPs which significantly associated with potential confounders. After excluding 32 SNPs associated with potential confounders, we repeated our main analyses using the remaining SNPs. (5) Although all 53 SNPs were the lead insulin-associated SNP at each 1 Mb region and all the SNPs were in different genomic regions, there may still be SNPs in linkage disequilibrium (LD). Therefore, we used LD clumping at a threshold of r^2^ < 0.001 (clumping window: 10,000 kB) to minimize the potential bias in effect estimates induced by the correlation between SNPs. We conducted an additional analysis using these independent SNPs as instrumental variables for IR.

As a widely measured marker of IR, the inclusion of fasting insulin in our study can provide support in the inference of the causal association between genetically predicted IR and ventricular structure and function. We selected genetic variants for fasting insulin from a large GWAS data, which included 108,557 individuals of European ancestry without diabetes (known as MAGIC) [12]. We only selected SNPs reaching genome-wide significance level (*P* < 5 × 10^−8^). After LD clumping at a threshold of r^2^ < 0.001 (clumping window: 10,000 kB), we identified 16 SNPs for fasting insulin. The genetic variants selection for HbA1c and fasting glucose levels was based on two large GWAS meta-analyses, which included 123,665 and 133,010 individuals of European ancestry without diabetes, respectively (known as MAGIC) [11, 12]. We identified 34 SNPs for HbA1c and 33 SNPs for fasting glucose at genome-wide significance level of *P* < 5 × 10^−8^ after LD clumping. Additional file 2: Tables S2–S5 present the detailed information of SNPs used as instrumental variables.

### Outcome sources

Cardiac imaging, such as cardiovascular magnetic resonance (CMR), is an important and widely used tool for determining ventricular structure and function [29]. For the LV parameters, we used summary data from the UK Biobank CMR study [13]. This GWAS was conducted on 16,923 European individuals without prevalent myocardial infarction or heart failure. The relevant LV parameters were obtained by CMR measurement. In this study, they performed GWASs on six LV parameters, including LV end-diastolic volume, LV end-systolic volume, LV stroke volume, LV ejection fraction, LV mass, and LV mass-to-end-diastolic volume ratio. Five summary data (except LV stroke volume) of LV parameters were available and served as the outcomes of our study. We used the selected SNPs of exposures to extract information from these summary data of LV parameters. For IR phenotypes, rs8101064 cannot be extracted from the outcome dataset, leaving 52 SNPs as instrumental variables for IR. For HbA1c, rs6474359 cannot be extracted from the outcome dataset, and rs10774625 was removed because it was associated with LV end-diastolic volume at genome-wide significance level, leaving 33 SNPs as instrumental variables for HbA1c. For fasting glucose, rs16913693 and rs6113722 cannot be extracted in the outcome dataset, leaving 31 SNPs as instrumental variables for fasting glucose (Additional file 2: Tables S2–S5).

### Statistical analysis

All analyses were performed in R software (version 4.0.0; R Foundation for Statistical Computing, Vienna, Austria) using the “MendelianRandomization”, “TwoSampleMR”, and “MR-PRESSO” packages.

### Two-sample univariable mendelian randomization

The random effects inverse-variance weighted (IVW) approach was the main MR method in our study. Effect estimates and corresponding standard errors of the selected SNPs were extracted from glycemic traits GWAS summary data and LV parameters GWAS summary data. We performed harmonization of the direction of estimates by effect alleles, where palindromic SNPs were aligned when minor allele frequencies were less than 0.3, or they were otherwise excluded. Then, we used the Wald estimator to calculate the MR estimate for each instrument and the Delta method to calculate the standard error [30]. Finally, we combined individual MR estimates using IVW meta-analysis [30].

Given that the traditional IVW MR method is susceptible to unbalanced horizontal pleiotropy [24]. Thus, we used additional MR methods, such as weighted median [31], MR-Egger [15], and MR-multi-directional residual sum, and outliers (PRESSO) [32], to further verify causal effects. Regardless of the type of horizontal pleiotropy, the weighted median method will provide a robust estimate even only half of the SNPs meet the requirement of valid instruments [31]. The MR-Egger method can identify and control the bias due to directional pleiotropy. Even if all variants are invalid, the MR-Egger method will produce valid estimates as long as the associations of individual variants with the exposure are unrelated to the corresponding pleiotropic effects [15]. In addition, the intercept obtained from MR-Egger regression can be used as a measure of unbalanced pleiotropy (*P* < 0.05 indicated significance) [15]. Although the weighted median and MR-Egger methods are not as precise as the IVW method due to lower statistical power [33], the estimates will be more reliable if there are consistent results in the same direction across all those three methods [34]. MR-PRESSO method was used to detect and correct for any SNP outliers that reflected potentially pleiotropic biases. And MR-PRESSO also requires that at least half of the SNPs are valid instruments [32]. Finally, we conducted a further sensitivity analysis, including heterogeneity test and leave-one-out analysis. As a measure of overall pleiotropy, heterogeneity was examined by the modified Cochran’s Q statistic (*P* < 0.05 indicated significance) [15]. By removing each SNP in turn, the leave-one-out analysis could evaluate whether the MR estimate is driven or biased by potentially pleiotropic SNPs [35].

### Two-sample multivariable mendelian randomization

We performed a multivariate MR analysis to estimate the independent effects of IR and glycemic level on LV structure and function [36, 37]. Due to the limited available SNPs (total number of SNPs = 64,493) in the GWAS summary data of fasting glucose[12] (http://magicinvestigators.org/downloads/), most SNPs of IR and HbA1c cannot be extracted. Therefore, we only included IR and HbA1c as exposures in the multivariate MR analysis. First, we examined the bidirectional associations between genetically predicted IR and HbA1c by using univariable MR methods. Then, we conducted a multivariable MR analysis which included SNPs that were associated with either IR or HbA1c. An extension of the IVW MR method was used as the main analysis of the multivariable MR [38, 39]. In the sensitivity analyses, other multivariable MR methods, such as an extension of the weighted median and MR-Egger [39]. The MR-Egger intercept test was also used to account for the potential horizontal pleiotropy [15, 39]. To assess the heterogeneity of genetic variants, we used the Cochran heterogeneity test [15, 40]. In order to improving our understanding of glycemic traits as potential risk factors for heart failure, we further performed a multivariate MR analysis to estimate the independent effects of IR and HbA1c on the risk of heart failure. More detailed methods for multivariable MR analyses are available in Additional file 1: Method S1.

MR effect sizes were presented as the change in outcomes per 1-standard deviation (SD) increase in IR (55% higher fasting insulin adjusted for BMI, 0.89 mmol/l higher TGs, and 0.46 mmol/l lower HDL-C), fasting insulin (0.60 ln[pmol/l]), and fasting glucose (0.65 mmol/l) and per 1% increase in HbA1c levels. To account for multiple testing, we used a Bonferroni correction threshold of *P* < 0.0025 (α = 0.05/20, the number of inspections) as statistically significant evidence for a causal association, and considered *P*-values between 0.0025 and 0.05 as marginal significance associations in our analyses.

## Results

### Univariable mendelian randomization

**Fig. 1:**
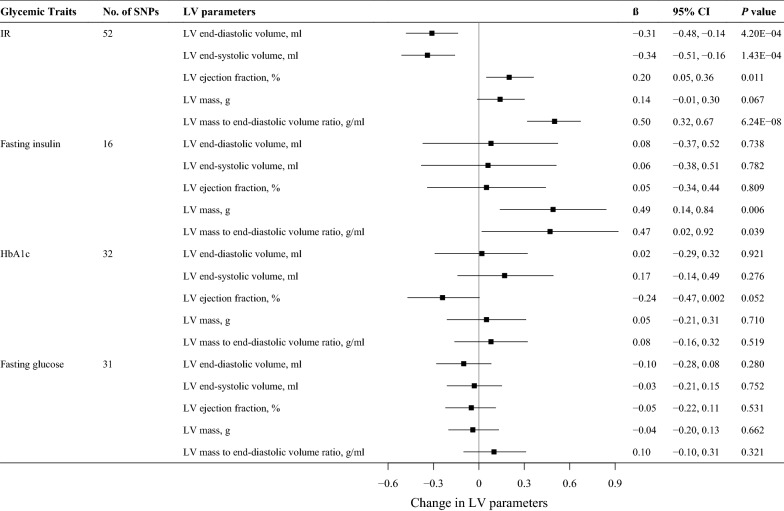
MR estimates of glycemic traits on left ventricular parameters. Estimates are derived from IVW MR analyses. Data are presented as change in LV parameter per 1-SD increase in insulin resistance (55% higher fasting insulin adjusted for BMI, 0.89 mmol/l higher TGs and 0.46 mmol/l lower HDL-C), fasting insulin (0.60 ln[pmol/l]), and fasting glucose (0.65 mmol/l) and per 1% increase in HbA1c levels. *IR* insulin resistance, *SNP* single nucleotide polymorphism, *LV* left ventricular, *HbA1c* hemoglobin A1c

For all analyses, there were no palindromic SNPs, so no extra SNPs were excluded from this analysis. Figure 1 shows the genetic association between glycemic traits and LV parameters. In the primary IVW MR analyses, per 1-SD increase in genetic liability to IR was significantly associated with lower LV end-diastolic volume (β = − 0.31 ml, 95% confidence interval [CI] − 0.48 to − 0.14 ml; *P* = 4.20 × 10^−4^), lower LV end-systolic volume (β = − 0.34 ml, 95% CI − 0.51 to − 0.16 ml; *P* = 1.43 × 10^−4^), and higher LV mass to end-diastolic volume ratio (β = 0.50 g/ml, 95% CI 0.32 to 0.67 g/ml; *P* = 6.24 × 10^−8^) after Bonferroni adjustment. Genetic liability to IR was also associated with higher LV ejection fraction by suggestive evidence (β = 0.20%, 95% CI 0.05 to 0.36%; *P* = 0.011). In addition, per 1-SD increase in genetic liability to fasting insulin levels was marginally significantly associated with higher LV mass (β = 0.49 g, 95% CI 0.14 to 0.84 g; *P* = 0.006) and LV mass-to-end-diastolic volume ratio (β = 0.47 g/ml, 95% CI 0.02 to 0.92 g/ml; *P* = 0.039). We observed neither genetic liability to HbA1c nor genetic liability to fasting glucose to be associated with any LV parameters.

Additional file 1: Table S6 shows the detailed information of additional MR methods for sensitivity analyses. For all analyses, the results from the weighted median and MR-Egger method supported a similar association. Due to lower statistical power, most estimates from these two methods were attenuated, but the direction of the results was consistent with the main IVW method. The scatterplots of SNP effects on glycemic traits versus their effects on LV parameters are displayed in Additional file 1: Figs. S1–S5. Several sensitivity analyses showed evidence of heterogeneity (Additional file 1: Table S7) but no proof of unbalanced pleiotropy, as assessed by the *P* values of Egger-intercept (all *P* > 0.05). No significant outlier SNPs were identified through the MR-PRESSO method. In the leave-one-out analysis, the main results were similar after removing each SNP in turn, indicating that no single SNP had an undue influence on the overall causal effect estimate (Additional file 1: Figs. S6–S10).

**Fig. 2:**
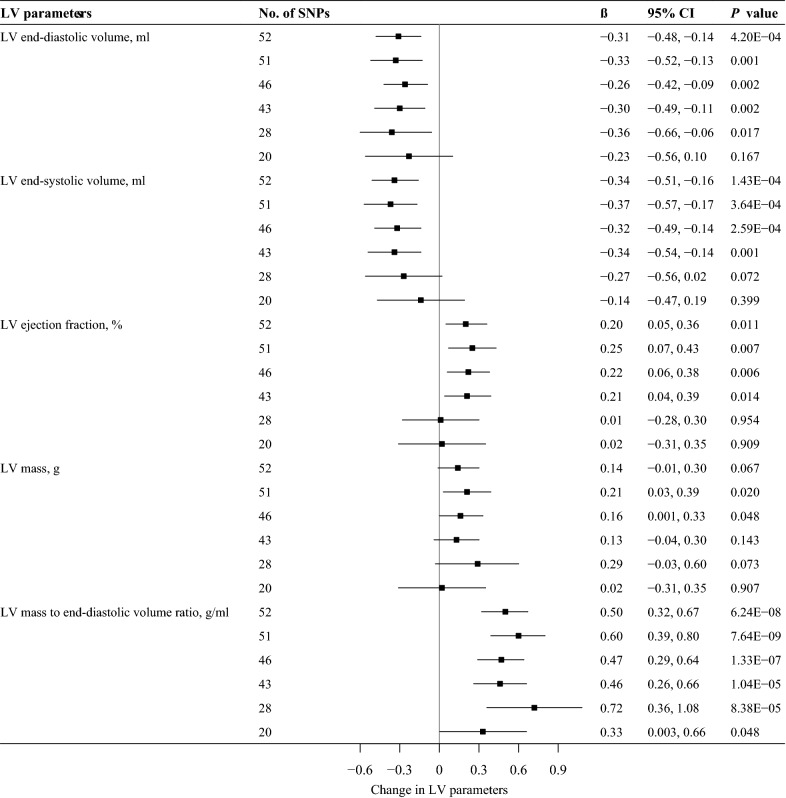
MR estimates of insulin resistance on left ventricular parameters. Estimates are derived from IVW MR analyses. 53-SNPs instruments were identified by Lotta et al., but rs8101064 cannot be extracted in the outcome dataset, so 52-SNPs instruments were used in the main analysis. 51-SNPs instruments with the exclusion of rs1011685 (near *LPL* gene). 46-SNPs instruments after linkage disequilibrium (LD) clumping at a threshold of r^2^ < 0.001 (clumping window: 10,000 kB). 43-SNPs instruments after exclusion of 9 SNPs individually associated with BMI at *P* < 0.001 using GIANT summary statistics. 28-SNPs instruments after exclusion of 25 SNPs (rs8101064 is one of these 25 SNPs) previously associated with triglycerides or high-density lipoprotein cholesterol at genome-wide significance. 20-SNPs instruments after exclusion of 32 SNPs previously associated with potential confounders at genome-wide significance. *SNP* single nucleotide polymorphism, *LV* left ventricular

In the additional analyses of IR, similar magnitudes of association were observed by using the 51-SNPs (excluding rs1011685 near *LPL*), 46-SNPs (after LD clumping), 43-SNPs (excluding genetic variants associated with BMI), 28-SNPs (excluding genetic variants associated with TGs or HDL-C) and 20-SNPs (excluding genetic variants associated with potential confounders) instruments (Fig. 2). After excluding rs1011685, genetic liability to IR was also significantly associated with lower LV end-diastolic volume (β = − 0.33 ml, 95% CI − 0.52 to − 0.13 ml; *P* = 0.001), lower LV end-systolic volume (β = − 0.37 ml, 95% CI − 0.57 to − 0.17 ml; *P* = 3.64 × 10^−4^), and higher LV mass to end-diastolic volume ratio (β = 0.60 g/ml, 95% CI 0.39 to 0.80 g/ml; *P* = 7.64 × 10^− 9^). When using 46-SNPs instruments, genetic liability to IR was still significantly associated with lower LV end-diastolic volume (β = − 0.26 ml, 95% CI − 0.42 to − 0.09 ml; *P* = 0.002), lower LV end-systolic volume (β = − 0.32 ml, 95% CI − 0.49 to − 0.14 ml; *P* = 2.89 × 10^−4^), and higher LV mass to end-diastolic volume ratio (β = 0.47 g/ml, 95% CI 0.29 to 0.64 g/ml; *P* = 1.31 × 10^−7^), although the magnitude of the effect was reduced. When using 43-SNPs and 28-SNPs instruments, genetic liability to IR was still significantly associated with LV mass-to-end-diastolic volume ratio. Using 20-SNPs instruments, genetic liability to IR was also marginally significantly associated with higher LV mass to end-diastolic volume ratio. Similar estimates were derived using additional MR methods although most estimates were attenuated (Additional file 1: Tables S8, S9).

### Multivariable mendelian randomization

In the bidirectional MR analyses, per 1-SD increase in genetic liability to IR was significantly associated with higher HbA1c (β = 0.06%, 95% CI 0.02 to 0.10%; P = 9.60 × 10^−4^), whereas genetic liability to HbA1c did not result in significant alterations in IR (Additional file 1: Tables S10, S11 and Figs. S11, S12). When both IR and HbA1c were included in a single multivariate model, there was evidence that per 1-SD increase in genetic liability to IR was independently associated with lower LV end-diastolic volume (β = − 0.27 ml, 95% CI − 0.47 to − 0.08 ml; *P* = 0.006), lower LV end-systolic volume (β = − 0.31 ml, 95% CI − 0.51 to − 0.11 ml; *P* = 0.002), higher LV ejection fraction by suggestive evidence (β = 0.19%, 95% CI 0.03 to 0.35%; *P* = 0.022) and higher LV mass to end-diastolic volume ratio (β = 0.48 g/ml, 95% CI 0.30 to 0.65 g/ml; *P* = 6.24 × 10^−8^). No evidence supported that genetic liability to HbA1c was associated with LV structure and function (Table 2). The effect estimates from multivariable MR-Egger and weighted median were similar to the IVW analysis (Table 2). Several sensitivity analyses showed evidence of potential heterogeneity and directional pleiotropy (Additional file 1: Table S12). In addition, our results demonstrated strong evidence for an independent effect of IR on heart failure (OR = 1.24; 95% CI 1.02 to 1.50; *P* = 0.031) (Additional file 1: Tables S13, S14).

**Table 2:** Independent effects of insulin resistance and hemoglobin A1c on left ventricular parameters assessed by multivariate MR

	IVW		Weighted-median		MR-Egger	
	β (95% CI)	*P*-value	β (95% CI)	*P*-value	β (95% CI)	*P*-value
LV end-diastolic volume, ml						
IR	− 0.27 (− 0.47, − 0.08)	0.006	− 0.25 (− 0.48, − 0.01)	0.038	− 0.32 (− 0.61, − 0.03)	0.030
HbA1c	− 0.06 (− 0.34, 0.21)	0.647	0.01 (− 0.34, 0.36)	0.943	− 0.07 (− 0.34, 0.21)	0.643
LV end-systolic volume, ml						
IR	− 0.31 (− 0.51, − 0.11)	0.002	− 0.28 (− 0.52, − 0.03)	0.027	− 0.38 (− 0.67, − 0.08)	0.012
HbA1c	0.11 (− 0.17, 0.39)	0.437	0.16 (− 0.20, 0.51)	0.392	0.11 (− 0.17, 0.39)	0.445
LV ejection fraction, %						
IR	0.19 (0.03, 0.35)	0.022	0.06 (− 0.17, 0.29)	0.619	0.27 (0.03, 0.51)	0.026
HbA1c	− 0.23 (− 0.46, 0.003)	0.053	− 0.26 (− 0.59, 0.06)	0.113	− 0.23 (− 0.46, 0.01)	0.055
LV mass, g						
IR	0.15 (− 0.03, 0.33)	0.093	0.01 (− 0.25, 0.27)	0.935	− 0.04 (− 0.30, 0.22)	0.748
HbA1c	0.03 (− 0.23, 0.28)	0.827	0.02 (− 0.30, 0.34)	0.915	0.02 (− 0.22, 0.27)	0.851
LV mass to end-diastolic volume ratio, g/ml						
IR	0.48 (0.30, 0.65)	7.98 × 10^−8^	0.62 (0.35, 0.89)	5.79 × 10^−6^	0.29 (0.03, 0.54)	0.024
HbA1c	0.15 (− 0.09, 0.40)	0.223	0.08 (− 0.25, 0.41)	0.634	0.15 (− 0.09, 0.39)	0.226

*IR* insulin resistance, *HbA1c* hemoglobin A1c, *LV* left ventricular, *IVW* inverse-variance weighted, *CI* confidence interval, *MR* Mendelian randomization. In multivariate MR analysis of LV parameters, rs8101064 and rs6474359 cannot be extracted from the summary data of LV parameters and rs2943645 cannot be extracted from the summary data of HbA1c. In addition, rs10774625 was removed because it was associated with LV end-diastolic volume at genome-wide significance level. After LD clumping at a threshold of r^2^ < 0.001 (clumping window: 10,000 kB), 71 independent SNPs were used in the analysis

## Discussion

In this MR study, we found that genetic liability to IR was associated with adverse changes in LV structure and function. Whereas genetic liability to HbA1c or fasting glucose did not result in any significant alterations in LV parameters. Moreover, multivariable MR analyses provided evidence supported that genetic liability to IR is independently associated with adverse changes in LV parameters and risk of heart failure after controlling for HbA1c. Based on these findings, we concluded that genetic liability to IR but not glycemic level was associated with adverse changes in LV structure and function (Additional file 1: Fig. S13).

Previous observational studies have suggested that both IR and glycemic levels are associated with LV structure and function [41, 42]. In view of the strong associations of glycemic level with LV structure and function, lowering glucose level may help prevent heart failure. However, two recent meta-analyses, by summarizing data from randomized controlled trials, have shown that intensive glycemic control is unlikely to reduce but will increase instead, the risk of heart failure in patients with or at risk of type 2 diabetes [43, 44]. Our findings are consistent with the findings of randomized controlled trials, which do not recommend glycemic control as an effective strategy to prevent heart failure [43, 44]. Probably, dysglycemia alone does not explain changes in cardiac morphology in T2D patients, and hyperglycemia may lead to cardiovascular diseases via mechanisms other than LV remodeling [8, 45, 46]. Of note, the present study found that genetic liability to IR is related to changes in LV structure and function, which may strengthen our understanding of IR as a risk factor for heart failure by providing evidence of direct impact on cardiac morphology. Our result is also supported by a recent MR study, which confirmed the potential causal association between genetic liability to IR and heart failure [8].

Previous MR studies have also shown an association between individual IR-related traits (such as dyslipidemia and obesity) and ventricular structure and function [47, 48]. Therefore, one may concern that the potential association between genetic liability to IR and changes in LV structure and function as found by our study is contributed by pleiotropy of other IR-related traits or other potential confounders. However, genetic liability to IR was still significantly associated with LV mass-to-end-diastolic volume ratio after excluding genetic variants associated with lipids (using 28 SNPs for IR), BMI (using 43 SNPs for IR), and potential confounders (using 20 SNPs for IR). Therefore, the association of genetic liability to IR with LV structure and function is not likely confounded by pleiotropy of lipids or BMI.

In this study, genetic liability to IR was associated with higher LV ejection fraction, but limited LV expansion during diastole and systolic, indicating that systolic function was preserved and that diastolic function was reduced. This finding is in concert with previous cross-sectional studies, which suggested that IR is associated with diastolic dysfunction, even prior to diabetes development [41, 49]. In addition, a previous study conducted by Savji et al. [50] has also suggested that IR confers a higher risk of future heart failure with preserved ejection fraction (HFpEF), but not heart failure with reduced ejection fraction (HFrEF). Diabetes is associated with an increased risk of morbidity and mortality in patients with chronic HFpEF, and approximately 45% of HFpEF patients have diabetes [51]. Our results emphasize that IR is related to poor ventricular structure and deterioration of diastolic function, which may play a key role in the mechanism of HFpEF caused by diabetes [52].

Numerous experimental studies have proven that metabolic disturbances mediate the adverse effects of IR on LV [53–55]. Under stress states (such as pressure load, ischemia, or injury), IR prevents the energy source of cardiomyocytes switch from oxidation of free fatty acid to the more energy-efficient glycolysis, which will limit the heart’s capacity for adaptive energy response [54]. The compensatory increase in free fatty acid metabolism will lead to increased oxygen consumption, decreased cardiac efficiency, and lipotoxicity, resulting in further damage of cardiac structure [54]. Previous study suggested that IR was also related to dyslipidemia [56]. In the current study, we found that the association between genetic liability to IR and changes in LV structure and function was attenuated when genetic variants associated with lipids (using 28 SNPs for IR) were excluded, suggesting that dyslipidemia may be a downstream mediator of adverse LV structure and function caused by IR [47]. In addition, the effect of IR on changes in LV structure and function may be partially mediated by hyperinsulinemia [53, 55]. Cardiomyocytes are typical insulin-targeted cells, and hyperinsulinemia caused by IR will have a direct nutritional effect on the myocardium, that is, increases the mass of myocardium and decreases the cardiac output [55].

Although IR and hyperglycosemia are highly correlated with each other, our study has disentangled the complex relationship of IR and hyperglycosemia with changes in LV structure and function and has several clinical implications. First, our findings suggest that adverse changes in LV structure and function are the potential underlying pathophysiology on the effect of IR on heart failure as found by other studies [8]. Second, as IR is a modifiable factor, our findings suggest that the improvement of IR may be beneficial in preventing heart failure [57]. Third, when choosing antidiabetic medications, one should select medications, such as metformin, that could improve insulin sensitivity [6, 58]. In addition, sodium-glucose cotransporter 2 inhibitors, such as dapagliflozin, should also be selected to prevent the risk of heart failure [59–61]. A recent randomized controlled trial confirmed that dapagliflozin can improve ventricular remodeling in diabetic patients by improving IR [60].

There are several limitations to our study. First, due to a lack of large-scale GWAS data of insulin sensitivity, we only used 53 SNPs from a multi-trait GWAS as instrumental variables of IR trait [9]. This GWSA contains three traits of the IR phenotype (high fasting insulin levels adjusted for BMI, high TG levels, and low HDL-C), which is a proxy measure rather than a direct measure of IR. However, the genetic risk score based on these 53 SNPs has been confirmed to be related to the gold standard measures of IR in independent samples from the Fenland study and the other four cohorts [9, 19]. Second, although all 53 SNPs were the lead insulin-associated SNP at each 1 Mb region and all the SNPs were in different genomic regions, there may still be SNPs in LD. However, when using 46 independent SNPs as instruments, genetic liability to IR was still significantly associated with LV parameters. Third, several MR analyses performed in this study were heterogeneity and directional pleiotropy. Although the results obtained by several MR methods were similar, we cannot completely rule out the possibility of bias in the estimation of derived effects due to pleiotropic effects of genetic instruments. Fourth, selection bias may still be present in this study. In particular, the instruments for IR were derived from a meta-GWAS that included fasting insulin adjusted for BMI. Adjustment for BMI in discovery GWAS may lead to collider bias [62], as evidenced by the negative association of the instruments with BMI [9]. However, this negative association with BMI would be expected to reduce, rather than increase, the association of genetic liability to IR with LV parameters, and therefore is unlikely to cause a significant bias in our MR estimates. Furthermore, exclusion of genetic variants related to BMI (using 43 SNPs for IR) also suggested that our MR estimates did not change materially. Fifth, participants of the GWASs used in present study were mostly middle to older adults [9–13], and there was a time lag between genetic randomization (at conception) and genetic studies of disease outcomes in middle to old age [63]. Therefore, the effect of selective survival needs to be considered when interpreting our results. Finally, our analyses were conducted using GWAS summary data from European ancestry, which reduced the bias caused by population stratification but also made the causal inference of this study may be inapplicable to other ethnicities.

## Conclusions

In conclusion, our results suggest that genetic liability to IR is associated with adverse changes in LV parameters and heart failure risk. However, no evidence supporting the direct impact of genetic liability to glycemic level on LV structure and function was found. Our findings may strengthen our understanding of IR as a risk factor for heart failure by providing evidence of direct impact on cardiac morphology.

## Supplementary Information

**Additional file 1: Method S1.** The detailed procedure and sensitive analyses of the Multivariable MR analysis. **Table S1.** Insulin resistance-related SNPs associated with potential confounders. **Table S6.** Sensitivity MR analysis of the association between glycemic traits and left ventricular parameters. **Table S7.** Heterogeneity and horizontal pleiotropy test of the associations between glycemic traits and left ventricular parameters. **Table S8.** Additional MR analysis of the association between insulin resistance and left ventricular parameters. **Table S9.** Heterogeneity and horizontal pleiotropy test of the associations between insulin resistance and left ventricular parameters. **Table S10.** The bidirectional associations between insulin resistance and hemoglobin A1c. **Table S11.** Heterogeneity and horizontal pleiotropy text of the associations between insulin resistance and hemoglobin A1c. **Table S12.** Heterogeneity and horizontal pleiotropy text of the associations between insulin resistance, hemoglobin A1c and left ventricular parameters in multivariate MR Analyses. **Table S13.** Effects of insulin resistance and hemoglobin A1c on heart failure in univariable and multivariate MR Analyses. **Table S14.** Heterogeneity and horizontal pleiotropy text of the associations between insulin resistance, hemoglobin A1c and heart failure in univariable and multivariate MR Analyses. **Figure S1.** Scatterplots of the causal estimates of glycemic traits and left ventricular end-diastolic volume. **Figure S2.** Scatterplots of the causal estimates of glycemic traits and left ventricular end-systolic volume. **Figure S3.** Scatterplots of the causal estimates of glycemic traits and left ventricular ejection fraction. **Figure S4.** Scatterplots of the causal estimates of glycemic traits and left ventricular mass. **Figure S5.** Scatterplots of the causal estimates of glycemic traits and left ventricular mass to end-diastolic volume ratio. **Figure S6.** Leave-one-out analyses of the association between glycemic traits and left ventricular end-diastolic volume. **Figure S7.** Leave-one-out analyses of the association between glycemic traits and left ventricular end-systolic volume. **Figure S8.** Leave-one-out analyses of the association between glycemic traits and left ventricular ejection fraction. **Figure S9.** Leave-one-out analyses of the association between glycemic traits and left ventricular mass. **Figure S10.** Leave-one-out analyses of the association between glycemic traits and left ventricular mass to end-diastolic volume ratio. **Figure S11.** Scatterplots of the bidirectional association of IR and HbA1c. **Figure S12.** Leave-one-out analyses of the bidirectional association of IR and HbA1c. **Figure S13.** A brief schematic diagram of possible assumption of the association of IR and HbA1c with cardiac morphology.

**Additional file 2: Table S2.** Characteristics of the single-nucleotide polymorphisms associated with insulin resistance and their associations with left ventricular parameters. **Table S3.** Characteristics of the single-nucleotide polymorphisms associated with fasting insulin and their associations with left ventricular parameters. **Table S4.** Characteristics of the single-nucleotide polymorphisms associated with glycated hemoglobin and their associations with left ventricular parameters. **Table S5.** Characteristics of the single-nucleotide polymorphisms associated with fasting glucose and their associations with left ventricular parameters.

## Data Availability

Data on LV parameters heart failure can be downloaded from https://www.ebi.ac.uk/gwas/. Data on fasting insulin and HbA1c can be downloaded from http://magicinvestigators.org/. Data on TG and HDL-C can be downloaded from http://csg.sph.umich.edu//abecasis/public/lipids2013/.

## Abbreviations

BMI: Body mass index
CI: Confidence interval
GWAS: Genome-wide association study
HbA1c: Hemoglobin A1c
HDL-C: High-density lipoprotein cholesterol
HFpEF: Heart failure with preserved ejection fraction
HFrEF: Heart failure with reduced ejection fraction
IR: Insulin resistance
IVW: Inverse-variance weighted
LV: Left ventricular
MR: Mendelian randomization
SNP: Single nucleotide polymorphism
SD: Standard deviation
TG: Triglyceride
