# Supplementary material for: Effects of glycemic traits on left ventricular structure and function: a mendelian randomization study
Source: Cardiovasc Diabetol. 2022 Jun 17;21:109. doi: 10.1186/s12933-022-01540-6 (PMC9206364; doi:10.1186/s12933-022-01540-6)
Supplement: Supplementary file 1 — Additional file 1: Method S1. The detailed procedure and sensitive analyses of the Multivariable MR analysis. Table S1. Insulin resistance-related SNPs associated with potential confounders. Table S6. Sensitivity MR analysis of the association between glycemic traits and left ventricular parameters. Table S7. Heterogeneity and horizontal pleiotropy test of the associations between glycemic traits and left ventricular parameters. Table S8. Additional MR analysis of the association between insulin resistance and left ventricular parameters. Table S9. Heterogeneity and horizontal pleiotropy test of the associations between insulin resistance and left ventricular parameters. Table S10. The bidirectional associations between insulin resistance and hemoglobin A1c. Table S11. Heterogeneity and horizontal pleiotropy text of the associations between insulin resistance and hemoglobin A1c. Table S12. Heterogeneity and horizontal pleiotropy text of the associations between insulin resistance, hemoglobin A1c and left ventricular parameters in multivariate MR Analyses. Table S13. Effects of insulin resistance and hemoglobin A1c on heart failure in univariable and multivariate MR Analyses. Table S14. Heterogeneity and horizontal pleiotropy text of the associations between insulin resistance, hemoglobin A1c and heart failure in univariable and multivariate MR Analyses. Figure S1. Scatterplots of the causal estimates of glycemic traits and left ventricular end-diastolic volume. Figure S2. Scatterplots of the causal estimates of glycemic traits and left ventricular end-systolic volume. Figure S3. Scatterplots of the causal estimates of glycemic traits and left ventricular ejection fraction. Figure S4. Scatterplots of the causal estimates of glycemic traits and left ventricular mass. Figure S5. Scatterplots of the causal estimates of glycemic traits and left ventricular mass to end-diastolic volume ratio. Figure S6. Leave-one-out analyses of the association between glycemic traits and [file 12933_2022_1540_MOESM1_ESM.docx]

**Method S1.** **The detailed procedure and sensitive analyses of the Multivariable MR analysis.**

First, we performed a bidirectional MR analysis to assess the causal association between genetically predicted IR and HbA1c. We used the IR-related SNPs as exposure and extracted information from the outcome data of HbA1c [1]. Due to the lack of summary data of IR phenotype, we were unable to directly extract the information of HbA1c-related SNPs [2, 3]. However, we can calculate the beta values and standard errors of HbA1c-related SNPs by referring to the method of Wang et al., [3]. We first extracted the information of HbA1c-related SNPs association with three components of IR phenotype from GWAS summary data (fasting insulin adjusted for BMI from the MAGIC consortia[4, 5]; TGs and HDL-C TGs and HDL-C from the GLGC consortia[6]). For fasting insulin, we used metabochip association results when available[4] and standardized the beta coefficients of fasting insulin using the standard deviation (≈0.5882) in 8,917 participants of the Fenland study inconsistent with the previous study[3]. We aligned alleles across the three phenotypes such that the effect allele was the HbA1c-raising allele. The results of the meta-analysis (using the fixed-effects inverse variance method) were the beta coefficient of the HbA1c-related SNPs and served as the outcome data of IR phenotype.

Second, we performed a multivariable MR analysis to investigate the independent effects of IR and HbA1c on LV parameters by using a “MendelianRandomization v0.5.0” package in R[7]. An extension of the IVW MR method was served as the main analysis [7, 8]. Other multivariable MR methods, such as an extension of the weighted median and MR-Egger[7] were used as the sensitivity analyses. We used the MR-Egger intercept test [7, 9] and the Cochran heterogeneity test [9, 10] to account for the potential horizontal pleiotropy and heterogeneity of genetic variants, respectively.

In a recent MR study conducted by Mordi et al., [11], genetic liability to IR is associated with an increased risk of heart failure, whereas no significant associations were found between HbA1c or fasting glucose with heart failure risk. However, whether IR is still independently associated with heart failure after adjusting for glucose levels remains unclear. Therefore, we further performed a multivariate MR analysis to assess the independent effects of IR and HbA1c on the risk of heart failure. The summary data for heart failure were acquired from a genome-wide association study, which comprised 47309 cases and 930014 controls of European ancestry across 26 studies from the HERMES Consortium [12] inconsistent with Mordi et al., study[11]. We first conducted a univariable MR analysis and then examined the independent effects of IR and HbA1c on heart failure by using multivariate MR methods as described in the previous text.

**References**

1. Wheeler E, Leong A, Liu CT, Hivert MF, Strawbridge RJ, Podmore C *et al*. Impact of common genetic determinants of Hemoglobin A1c on type 2 diabetes risk and diagnosis in ancestrally diverse populations: A transethnic genome-wide meta-analysis. PLoS Med. 2017; 14:e1002383.

2. Lotta LA, Gulati P, Day FR, Payne F, Ongen H, van de Bunt M *et al*. Integrative genomic analysis implicates limited peripheral adipose storage capacity in the pathogenesis of human insulin resistance. Nat Genet. 2017; 49:17-26.

3. Wang Q, Holmes MV, Davey Smith G, Ala-Korpela M. Genetic Support for a Causal Role of Insulin Resistance on Circulating Branched-Chain Amino Acids and Inflammation. Diabetes Care. 2017; 40:1779-1786.

4. Scott RA, Lagou V, Welch RP, Wheeler E, Montasser ME, Luan J *et al*. Large-scale association analyses identify new loci influencing glycemic traits and provide insight into the underlying biological pathways. Nat Genet. 2012; 44:991-1005.

5. Manning AK, Hivert MF, Scott RA, Grimsby JL, Bouatia-Naji N, Chen H *et al*. A genome-wide approach accounting for body mass index identifies genetic variants influencing fasting glycemic traits and insulin resistance. Nat Genet. 2012; 44:659-669.

6. Willer CJ, Schmidt EM, Sengupta S, Peloso GM, Gustafsson S, Kanoni S *et al*. Discovery and refinement of loci associated with lipid levels. Nat Genet. 2013; 45:1274-1283.

7. Broadbent JR, Foley CN, Grant AJ, Mason AM, Staley JR, Burgess S. MendelianRandomization v0.5.0: updates to an R package for performing Mendelian randomization analyses using summarized data. Wellcome Open Res. 2020; 5:252.

8. Yavorska OO, Burgess S. MendelianRandomization: an R package for performing Mendelian randomization analyses using summarized data. Int J Epidemiol. 2017; 46:1734-1739.

9. Bowden J, Davey Smith G, Burgess S. Mendelian randomization with invalid instruments: effect estimation and bias detection through Egger regression. Int J Epidemiol. 2015; 44:512-525.

10. Bowden J, Del Greco MF, Minelli C, Zhao Q, Lawlor DA, Sheehan NA, Thompson J, Davey Smith G. Improving the accuracy of two-sample summary-data Mendelian randomization: moving beyond the NOME assumption. Int J Epidemiol. 2019; 48:728-742.

11. Mordi IR, Lumbers RT, Palmer CNA, Pearson ER, Sattar N, Holmes MV, Lang CC, Consortium H. Type 2 Diabetes, Metabolic Traits, and Risk of Heart Failure: A Mendelian Randomization Study. Diabetes Care. 2021.

12. Shah S, Henry A, Roselli C, Lin H, Sveinbjornsson G, Fatemifar G *et al*. Genome-wide association and Mendelian randomisation analysis provide insights into the pathogenesis of heart failure. Nat Commun. 2020; 11:163.

**Table S1. Insulin resistance-related SNPs associated with potential confounders**

| **SNP** | **Potential confounders** |
| --- | --- |
| rs9425291 | Waist to hip ratio |
| rs4846565 | Obesity-related traits |
| rs2249105 | Systolic blood pressure |
| rs10195252 | Obesity-related traits |
| rs492400 | BMI |
| rs2943645 | Obesity-related traits |
| rs308971 | BMI |
| rs295449 | inflammatory cells |
| rs11130329 | Pulse rate, obesity-related traits |
| rs645040 | Obesity-related traits, coronary artery disease |
| rs2699429 | Pulmonary function |
| rs3822072 | Obesity-related traits |
| rs459193 | Obesity-related traits |
| rs4976033 | Obesity-related traits |
| rs1045241 | Waist to hip ratio |
| rs2434612 | Obesity-related traits |
| rs966544 | Diastolic blood pressure, waist to hip ratio |
| rs12525532 | Obesity-related traits |
| rs6937438 | log eGFR creatinine, serum urate |
| rs2745353 | Obesity-related traits |
| rs9492443 | Obesity-related traits, pulmonary function |
| rs972283 | Obesity-related traits, hypertension |
| rs2126259 | C reactive protein levels, inflammatory cells |
| rs1011685 | Coronary artery disease |
| rs4738141 | Waist to hip ratio |
| rs11231693 | Waist to hip ratio |
| rs718314 | Waist to hip ratio |
| rs7973683 | Obesity-related traits |
| rs8032586 | Pulse rate |
| rs4804833 | Obesity-related traits |
| rs731839 | Obesity-related traits |
| rs132985 | Obesity-related traits |

Using the PhenoScanner tool (http://www.phenoscanner.medschl.cam.ac.uk/), we assessed SNPs at a threshold of *P* < 5×10^-8^ for their association with other potential confounders, which may affect ventricular structure and function. SNP, single nucleotide polymorphism; BMI, body mass index; GFR, glomerular filtration rate.

**Table S6.** Sensitivity MR analysis of the association between glycemic traits and left ventricular parameters

|  | **IVW** | | **Weighted-median** | | **MR-Egger** | | ***MR-PRESSO** |
| --- | --- | --- | --- | --- | --- | --- | --- |
|  | **β±SE** | ***P-value*** | **β±SE** | ***P-value*** | **β±SE** | ***P-value*** |  |
| **IR** |  |  |  |  |  |  |  |
| LV end-diastolic volume, ml | -0.31±0.09 | 4.20×10^-4^ | -0.25±0.13 | 0.060 | -0.26±0.18 | 0.156 | --- |
| LV end-systolic volume, ml | -0.34±0.09 | 1.43×10^-4^ | -0.25±0.14 | 0.062 | -0.36±0.18 | 0.051 | --- |
| LV ejection fraction, % | 0.20±0.08 | 0.011 | 0.08±0.13 | 0.541 | 0.29±0.16 | 0.081 | --- |
| LV mass, g | 0.14±0.08 | 0.067 | -0.03±0.14 | 0.820 | -0.04±0.16 | 0.783 | --- |
| LV mass to end-diastolic volume ratio, g/ml | 0.50±0.09 | 6.24×10^-8^ | 0.25±0.12 | 0.037 | 0.20±0.18 | 0.266 | --- |
| **Fasting insulin** |  |  |  |  |  |  |  |
| LV end-diastolic volume, ml | 0.08±0.22 | 0.738 | 0.05±0.25 | 0.848 | 1.39±1.12 | 0.238 | --- |
| LV end-systolic volume, ml | 0.06±0.23 | 0.782 | -0.03±0.27 | 0.916 | 0.22±1.20 | 0.854 | --- |
| LV ejection fraction, % | 0.05±0.20 | 0.809 | 0.02±0.25 | 0.924 | 1.45±0.97 | 0.156 | --- |
| LV mass, g | 0.49±0.18 | 0.006 | 0.36±0.25 | 0.153 | 0.71±0.93 | 0.460 | --- |
| LV mass to end-diastolic volume ratio, g/ml | 0.47±0.23 | 0.039 | 0.12±0.26 | 0.640 | -1.56±1.06 | 0.165 | --- |
| **HbA1c** |  |  |  |  |  |  |  |
| LV end-diastolic volume, ml | 0.02±0.16 | 0.921 | 0.05±0.18 | 0.786 | 0.02±0.29 | 0.949 | --- |
| LV end-systolic volume, ml | 0.17±0.16 | 0.276 | 0.17±0.18 | 0.348 | 0.21±0.30 | 0.487 | --- |
| LV ejection fraction, % | -0.24±0.12 | 0.052 | -0.26±0.17 | 0.129 | -0.41±0.22 | 0.078 | --- |
| LV mass, g | 0.05±0.13 | 0.710 | 0.001±0.17 | 0.997 | 0.02±0.25 | 0.935 | --- |
| LV mass to end-diastolic volume ratio, g/ml | 0.08±0.12 | 0.519 | 0.09±0.19 | 0.618 | 0.13±0.23 | 0.582 | --- |
| **Fasting glucose** |  |  |  |  |  |  |  |
| LV end-diastolic volume, ml | -0.10±0.09 | 0.280 | -0.05±0.12 | 0.655 | -0.17±0.17 | 0.321 | --- |
| LV end-systolic volume, ml | -0.03±0.09 | 0.752 | 0.06±0.12 | 0.604 | -0.12±0.17 | 0.479 | --- |
| LV ejection fraction, % | -0.05±0.09 | 0.531 | -0.10±0.11 | 0.368 | -0.02±0.16 | 0.888 | --- |
| LV mass, g | -0.04±0.08 | 0.662 | 0.03±0.10 | 0.810 | 0.02±0.15 | 0.882 | --- |
| LV mass to end-diastolic volume ratio, g/ml | 0.10±0.11 | 0.321 | 0.11±0.11 | 0.313 | 0.34±0.19 | 0.076 | --- |

Data are presented as change in LV parameter per 1-SD increase in insulin resistance (55% higher fasting insulin adjusted for BMI, 0.89 mmol/L higher TGs and 0.46 mmol/L lower HDL-C), fasting insulin (0.60 ln[pmol/L]), and fasting glucose (0.65 mmol/L) and per 1% increase in HbA1c levels. LV, left ventricular; IR, insulin resistance; HbA1c, hemoglobin A1c; MR, Mendelian randomization; IVW, inverse-variance weighted; MR-PRESSO, MR-multi-directional residual sum and outliers; SE, standard error. *No significant outlier SNPs were found through the MR-PRESSO method.

**Table S7.** Heterogeneity and horizontal pleiotropy test of the associations between glycemic traits and left ventricular parameters.

|  | **Weak instruments** | **IVW** | | **MR-Egger** | | | | | | |
| --- | --- | --- | --- | --- | --- | --- | --- | --- | --- | --- |
|  | **Mean F-value** | **Cochran’s Q** | ***P-value*** | **Cochran’s Q** | ***P-value*** | **Intercept** | **SE** | ***P-value* for**  **intercept** | | |
| **IR** | | | | | |  |  |  |  |  |
| LV end-diastolic volume, ml | 89.27 | 62.25 | 0.134 | 62.12 | 0.117 | -0.001 | 0.004 | 0.751 | | |
| LV end-systolic volume, ml |  | 64.93 | 0.091 | 64.89 | 0.077 | 6.80×10^-4^ | 0.004 | 0.865 | | |
| LV ejection fraction, % |  | 44.23 | 0.738 | 43.86 | 0.717 | -0.002 | 0.004 | 0.549 | | |
| LV mass, g |  | 50.73 | 0.485 | 48.90 | 0.518 | 0.005 | 0.003 | 0.182 | | |
| LV mass to end-diastolic volume ratio, g/ml |  | 68.93 | 0.048 | 64.60 | 0.080 | 0.007 | 0.004 | 0.073 | | |
| **Fasting insulin** | | | | | |  |  |  |  |  |
| LV end-diastolic volume, ml | 42.62 | 23.96 | 0.066 | 21.76 | 0.084 | -0.022 | 0.019 | 0.254 | | |
| LV end-systolic volume, ml |  | 24.73 | 0.054 | 24.69 | 0.038 | -0.003 | 0.020 | 0.893 | | |
| LV ejection fraction, % |  | 18.39 | 0.243 | 15.90 | 0.320 | -0.024 | 0.016 | 0.161 | | |
| LV mass, g |  | 14.92 | 0.457 | 14.86 | 0.388 | -0.004 | 0.015 | 0.816 | | |
| LV mass to end-diastolic volume ratio, g/ml |  | 24.63 | 0.055 | 19.38 | 0.151 | 0.034 | 0.018 | 0.072 | | |
| **HbA1c** | | | | | |  |  |  |  |  |
| LV end-diastolic volume, ml | 80.66 | 58.92 | 0.002 | 58.92 | 0.001 | -8.27×10^-5^ | 0.006 | 0.989 | | |
| LV end-systolic volume, ml |  | 61.86 | 8.09×10^-4^ | 61.82 | 5.51×10^-4^ | -8.67×10^-4^ | 0.006 | 0.885 | | |
| LV ejection fraction, % |  | 35.16 | 0.277 | 34.19 | 0.273 | 0.004 | 0.004 | 0.362 | | |
| LV mass, g |  | 43.50 | 0.067 | 43.47 | 0.053 | 6.89×10^-4^ | 0.005 | 0.891 | | |
| LV mass to end-diastolic volume ratio, g/ml |  | 35.34 | 0.271 | 35.26 | 0.233 | -0.001 | 0.005 | 0.802 | | |
| **Fasting glucose** | | | | | |  |  |  |  |  |
| LV end-diastolic volume, ml | 132.19 | 42.70 | 0.062 | 42.33 | 0.052 | 0.002 | 0.005 | 0.617 | | |
| LV end-systolic volume, ml |  | 45.14 | 0.037 | 44.49 | 0.033 | 0.003 | 0.005 | 0.518 | | |
| LV ejection fraction, % |  | 36.49 | 0.193 | 36.42 | 0.162 | -0.001 | 0.005 | 0.813 | | |
| LV mass, g |  | 34.56 | 0.259 | 34.31 | 0.228 | -0.002 | 0.004 | 0.646 | | |
| LV mass to end-diastolic volume ratio, g/ml |  | 56.16 | 0.003 | 51.95 | 0.006 | -0.008 | 0.005 | 0.136 | | |

IR, insulin resistance; HbA1c, hemoglobin A1c; LV, left ventricular; IVW, inverse-variance weighted.

**Table S8.** Additional MR analysis of the association between insulin resistance and left ventricular parameters

|  | **IVW** | | **Weighted-median** | | **MR-Egger** | | ***MR-PRESSO** |
| --- | --- | --- | --- | --- | --- | --- | --- |
|  | **β±SE** | ***P-value*** | **β±SE** | ***P-value*** | **β±SE** | ***P-value*** |  |
| **LV end-diastolic volume, ml** |  |  |  |  |  |  |  |
| 52-SNPs | -0.31±0.09 | 4.20×10^-4^ | -0.25±0.13 | 0.060 | -0.26±0.18 | 0.156 | --- |
| 51-SNPs | -0.33±0.10 | 0.001 | -0.32±0.14 | 0.022 | -0.32±0.36 | 0.372 | --- |
| 46- SNPs | -0.26±0.08 | 0.002 | -0.35±0.14 | 0.066 | -0.26±0.17 | 0.141 | --- |
| 43-SNPs | -0.30±0.10 | 0.002 | -0.25±0.14 | 0.080 | -0.28±0.19 | 0.147 | --- |
| 28-SNPs | -0.36±0.15 | 0.017 | -0.34±0.22 | 0.120 | -0.94±0.68 | 0.183 | --- |
| 20-SNPs | -0.23±0.17 | 0.167 | -0.21±0.23 | 0.356 | -0.83±0.61 | 0.192 | --- |
| **LV end-systolic volume, ml** |  |  |  |  |  |  |  |
| 52-SNPs | -0.34±0.09 | 1.43×10^-4^ | -0.25±0.14 | 0.062 | -0.36±0.18 | 0.051 | --- |
| 51-SNPs | -0.37±0.10 | 3.64×10^-4^ | -0.31±0.14 | 0.027 | -0.76±0.36 | 0.039 | --- |
| 46-SNPs | -0.32±0.09 | 2.89×10^-4^ | -0.25±0.14 | 0.070 | -0.33±0.18 | 0.068 | --- |
| 43-SNPs | -0.34±0.10 | 0.001 | -0.25±0.14 | 0.070 | -0.36±0.20 | 0.086 | --- |
| 28-SNPs | -0.27±0.15 | 0.072 | -0.15±0.21 | 0.479 | -1.02±0.66 | 0.138 | --- |
| 20-SNPs | -0.14±0.17 | 0.399 | -0.14±0.23 | 0.561 | -0.72±0.61 | 0.254 | --- |
| **LV ejection fraction, %** |  |  |  |  |  |  |  |
| 52-SNPs | 0.20±0.08 | 0.011 | 0.08±0.13 | 0.541 | 0.29±0.16 | 0.081 | --- |
| 51-SNPs | 0.25±0.09 | 0.007 | 0.22±0.13 | 0.099 | 1.00±0.32 | 0.003 | --- |
| 46-SNPs | 0.22±0.08 | 0.006 | 0.10±0.13 | 0.446 | 0.23±0.16 | 0.168 | --- |
| 43-SNPs | 0.21±0.09 | 0.014 | 0.07±0.14 | 0.642 | 0.25±0.17 | 0.146 | --- |
| 28-SNPs | 0.01±0.15 | 0.954 | -0.08±0.20 | 0.710 | 0.51±0.67 | 0.454 | --- |
| 20-SNPs | 0.02±0.17 | 0.909 | 0.04±0.23 | 0.867 | 0.60±0.61 | 0.345 | --- |
| **LV mass, g** |  |  |  |  |  |  |  |
| 52-SNPs | 0.14±0.08 | 0.067 | -0.03±0.14 | 0.820 | -0.04±0.16 | 0.783 | --- |
| 51-SNPs | 0.21±0.09 | 0.020 | 0.16±0.14 | 0.243 | 0.13±0.32 | 0.689 | --- |
| 46-SNPs | 0.17±0.08 | 0.048 | -0.03±0.13 | 0.821 | -0.06±0.17 | 0.718 | --- |
| 43-SNPs | 0.13±0.09 | 0.143 | -0.04±0.14 | 0.776 | -0.13±0.17 | 0.455 | --- |
| 28-SNPs | 0.29±0.16 | 0.073 | 0.15±0.21 | 0.460 | 0.39±0.73 | 0.597 | --- |
| 20-SNPs | 0.02±0.17 | 0.907 | 0.003±0.22 | 0.989 | -0.08±0.61 | 0.894 | --- |
| **LV mass to end-diastolic volume ratio, g/ml** |  |  |  |  |  |  |  |
| 52-SNPs | 0.50±0.09 | 6.24×10^-8^ | 0.25±0.12 | 0.037 | 0.20±0.18 | 0.266 | --- |
| 51-SNPs | 0.60±0.10 | 7.64×10^-9^ | 0.64±0.14 | 5.39×10^-6^ | 0.44±0.36 | 0.228 | --- |
| 46-SNPs | 0.47±0.09 | 1.31×10^-7^ | 0.21±0.13 | 0.095 | 0.18±0.17 | 0.300 | --- |
| 43-SNPs | 0.46±0.10 | 1.04×10^-5^ | 0.20±0.14 | 0.147 | 0.15±0.19 | 0.434 | --- |
| 28-SNPs | 0.72±0.18 | 8.38×10^-5^ | 0.57±0.22 | 0.010 | 1.18±0.83 | 0.165 | --- |
| 20-SNPs | 0.33±0.17 | 0.048 | 0.32±0.22 | 0.152 | 0.81±0.61 | 0.202 | --- |

53-SNPs instruments were identified by Lotta et al., but rs8101064 cannot be extracted in the outcome dataset, so 52-SNPs instruments were used in the main analysis. 51-SNPs instruments with the exclusion of rs1011685 (near *LPL* gene). 46-SNPs instruments After linkage disequilibrium (LD) clumping at a threshold of r^2^ < 0.001 (clumping window: 10000 kB). 43-SNPs instruments after exclusion of 9 SNPs individually associated with BMI at *P* < 0.001 using GIANT summary statistics. 28-SNPs instruments after exclusion of 25 SNPs (rs8101064 is one of these 25 SNPs) previously associated with triglycerides or high-density lipoprotein cholesterol at genome-wide significance. 20-SNPs instruments after exclusion of 32 SNPs previously associated with potential confounders at genome-wide significance. SNP, single nucleotide polymorphism; LV, left ventricular. MR, Mendelian randomization; MR-PRESSO, MR-multi-directional residual sum and outliers. *No significant outlier SNPs were found through the MR-PRESSO method.

**Table S9.** Heterogeneity and horizontal pleiotropy test of the associations between insulin resistance and left ventricular parameters.

|  | **Weak instruments** | **IVW** | | **MR-Egger** | | | | | | |
| --- | --- | --- | --- | --- | --- | --- | --- | --- | --- | --- |
|  | **Mean F-value** | **Cochran’s Q** | ***P-value*** | **Cochran’s Q** | ***P-value*** | **Intercept** | **SE** | ***P-value* for**  **intercept** | | |
| **52-SNPs** | | | | | |  |  |  |  |  |
| LV end-diastolic volume, ml | 89.27 | 62.25 | 0.134 | 62.12 | 0.117 | -0.001 | 0.004 | 0.751 | | |
| LV end-systolic volume, ml |  | 64.93 | 0.091 | 64.89 | 0.077 | 6.80×10^-4^ | 0.004 | 0.865 | | |
| LV ejection fraction, % |  | 44.23 | 0.738 | 43.86 | 0.717 | -0.002 | 0.004 | 0.549 | | |
| LV mass, g |  | 50.73 | 0.485 | 48.90 | 0.518 | 0.005 | 0.003 | 0.182 | | |
| LV mass to end-diastolic volume ratio, g/ml |  | 68.93 | 0.048 | 65.60 | 0.080 | 0.007 | 0.004 | 0.073 | | |
| **51-SNPs** | | | | | |  |  |  |  |  |
| LV end-diastolic volume, ml | 66.71 | 62.07 | 0.118 | 62.07 | 0.100 | -8.84×10^-5^ | 0.007 | 0.990 | | |
| LV end-systolic volume, ml |  | 64.47 | 0.082 | 62.80 | 0.089 | 0.008 | 0.007 | 0.260 | | |
| LV ejection fraction, % |  | 43.21 | 0.740 | 37.21 | 0.891 | -0.015 | 0.006 | 0.018 | | |
| LV mass, g |  | 48.58 | 0.530 | 48.50 | 0.493 | 0.002 | 0.006 | 0.784 | | |
| LV mass to end-diastolic volume ratio, g/ml |  | 64.10 | 0.087 | 63.85 | 0.075 | 0.003 | 0.007 | 0.661 | | |
| **46-SNPs** |  |  |  |  |  |  |  |  | | |
| LV end-diastolic volume, ml | 95.33 | 49.10 | 0.312 | 49.10 | 0.276 | -8.42×10^-6^ | 0.004 | 0.998 | | |
| LV end-systolic volume, ml |  | 51.85 | 0.224 | 51.84 | 0.195 | 3.61×10^-4^ | 0.004 | 0.928 | | |
| LV ejection fraction, % |  | 35.86 | 0.833 | 35.86 | 0.804 | -1.53×10^-4^ | 0.004 | 0.967 | | |
| LV mass, g |  | 48.37 | 0.338 | 45.78 | 0.398 | 0.006 | 0.004 | 0.121 | | |
| LV mass to end-diastolic volume ratio, g/ml |  | 53.85 | 0.172 | 49.65 | 0.258 | 0.007 | 0.004 | 0.060 | | |
| **43-SNPs** | | | | | |  |  |  |  |  |
| LV end-diastolic volume, ml | 87.43 | 53.54 | 0.109 | 53.52 | 0.091 | -3.97×10^-4^ | 0.004 | 0.925 | | |
| LV end-systolic volume, ml |  | 60.26 | 0.034 | 60.24 | 0.027 | 3.92×10^-4^ | 0.004 | 0.930 | | |
| LV ejection fraction, % |  | 40.32 | 0.545 | 40.26 | 0.503 | -8.72×10^-4^ | 0.004 | 0.813 | | |
| LV mass, g |  | 43.09 | 0.425 | 39.89 | 0.520 | 0.007 | 0.004 | 0.081 | | |
| LV mass to end-diastolic volume ratio, g/ml |  | 60.16 | 0.034 | 55.60 | 0.064 | 0.008 | 0.004 | 0.074 | | |
| **28-SNPs** | | | | | |  |  |  |  |  |
| LV end-diastolic volume, ml | 44.38 | 28.38 | 0.392 | 27.59 | 0.379 | 0.009 | 0.011 | 0.397 | | |
| LV end-systolic volume, ml |  | 26.22 | 0.506 | 24.88 | 0.526 | 0.012 | 0.011 | 0.257 | | |
| LV ejection fraction, % |  | 15.76 | 0.957 | 15.17 | 0.954 | -0.008 | 0.011 | 0.450 | | |
| LV mass, g |  | 31.24 | 0.261 | 31.22 | 0.220 | -0.002 | 0.012 | 0.885 | | |
| LV mass to end-diastolic volume ratio, g/ml |  | 40.82 | 0.043 | 40.31 | 0.036 | -0.008 | 0.013 | 0.569 | | |
| **20-SNPs** |  |  |  |  |  |  |  |  | | |
| LV end-diastolic volume, ml | 47.46 | 14.60 | 0.748 | 13.57 | 0.757 | 0.010 | 0.010 | 0.324 | | |
| LV end-systolic volume, ml |  | 13.63 | 0.805 | 12.66 | 0.811 | 0.010 | 0.010 | 0.338 | | |
| LV ejection fraction, % |  | 12.84 | 0.846 | 11.89 | 0.853 | -0.010 | 0.010 | 0.342 | | |
| LV mass, g |  | 7.63 | 0.990 | 7.60 | 0.984 | 0.002 | 0.010 | 0.864 | | |
| LV mass to end-diastolic volume ratio, g/ml |  | 15.40 | 0.697 | 14.75 | 0.679 | -0.008 | 0.010 | 0.429 | | |

IR, insulin resistance; LV, left ventricular; IVW, inverse-variance weighted.

**Table S10.** The bidirectional associations between insulin resistance and hemoglobin A1c.

|  | **IVW** | | **Weighted-median** | | **MR-Egger** | | ***MR-PRESSO** | |
| --- | --- | --- | --- | --- | --- | --- | --- | --- |
|  | **β (95% CI)** | ***P-value*** | **β (95% CI)** | ***P-value*** | **β (95% CI)** | ***P-value*** | **β (95% CI)** | ***P-value*** |
| **Exposures-Outcome** | |  |  |  |  |  |  |  |
| IR-HbA1c | 0.06(0.02, 0.10) | 9.60×10^-4^ | 0.04(-0.005, 0.08) | 0.086 | -0.004(-0.07, 0.06) | 0.907 | 0.10(0.06, 0.14) | 5.38×10^-7^ |
| HbA1c-IR | 0.01(-0.05, 0.07) | 0.747 | 0.01(-0.06, 0.09) | 0.741 | 0.04(-0.10, 0.17) | 0.596 | 3.29×10^-5^(-0.06, 0.06) | 0.999 |

IR as exposures, rs2943645 cannot be extracted from the summary data of HbA1c, and rs9881942 was removed because it was associated with HbA1c at genome-wide significance level. Therefore, a total of 51 SNPs for IR and 34 SNPs for HbA1c were included in the bidirectional MR analyses. *****MR-PRESSO detected one significant outlier (rs1011685) for IR-HbA1c and one significant outlier (rs7616006) for HbA1c-IR. IR, insulin resistance; HbA1c, hemoglobin A1c; IVW, inverse-variance weighted; CI, confidence interval; MR, Mendelian randomization; MR-PRESSO, MR-multi-directional residual sum and outliers.

**Table S11.** Heterogeneity and horizontal pleiotropy text of the associations between insulin resistance and hemoglobin A1c.

|  | **IVW** | | | **MR-Egger** | | | | |
| --- | --- | --- | --- | --- | --- | --- | --- | --- |
|  | **Cochran’s Q** | | ***P-value*** | **Cochran’s Q** | ***P-value*** | **Intercept** | **SE** | ***P-value* for**  **intercept** |
| **Exposure-Outcome** | |  |  |  |  |  |  |  |
| IR-HbA1c | 118.96 | | 1.50×10^-7^ | 107.80 | 2.65×10^-6^ | 0.002 | 7.61×10^-4^ | 0.029 |
| HbA1c-IR | 65.15 | | 7.06×10^-4^ | 64.77 | 5.33×10^-4^ | -5.42×10^-4^ | 0.001 | 0.665 |

IR, insulin resistance; HbA1c, hemoglobin A1c; IVW, inverse-variance weighted.

**Table S12.** Heterogeneity and horizontal pleiotropy text of the associations between insulin resistance, hemoglobin A1c and left ventricular parameters in multivariate MR Analyses

|  | **IVW** | | **MR-Egger** | | | | |
| --- | --- | --- | --- | --- | --- | --- | --- |
|  | **Cochran’s Q** | ***P-value*** | **Cochran’s Q** | ***P-value*** | **Intercept** | **SE** | ***P-value* for**  **intercept** |
| **LV end-diastolic volume, ml** |  |  |  |  |  |  |  |
| IR and HbA1c | 98.90 | 0.011 | 98.63 | 0.009 | 0.001 | 0.003 | 0.668 |
| **LV end-systolic volume, ml** |  |  |  |  |  |  |  |
| IR and HbA1c | 104.20 | 0.004 | 103.65 | 0.004 | 0.002 | 0.003 | 0.548 |
| **LV ejection fraction, %** |  |  |  |  |  |  |  |
| IR and HbA1c | 61.80 | 0.719 | 60.98 | 0.714 | -0.002 | 0.002 | 0.366 |
| **LV mass, g** |  |  |  |  |  |  |  |
| IR and HbA1c | 82.93 | 0.107 | 79.15 | 0.167 | 0.005 | 0.002 | 0.043 |
| **LV mass to end-diastolic volume ratio, g/ml** | | | | |  |  |  |
| IR and HbA1c | 79.58 | 0.180 | 74.95 | 0.263 | 0.005 | 0.002 | 0.040 |

IR, insulin resistance; HbA1c, hemoglobin A1c; IVW, inverse-variance weighted; LV, left ventricular.

**Table S13.** Effects of insulin resistance and hemoglobin A1c on heart failure in univariable and multivariate MR Analyses.

|  | **IVW** | | **Weighted-median** | | **MR-Egger** | | *******MR-PRESSO** | |
| --- | --- | --- | --- | --- | --- | --- | --- | --- |
|  | **OR (95%)** | ***P-value*** | **OR (95%)** | ***P-value*** | **OR (95%)** | ***P-value*** | **β (95%)** | ***P-value*** |
| **Univariable Mendelian randomization** | | |  |  |  |  |  |  |
| IR | 1.19(1.01, 1.41) | 0.042 | 1.31(1.08, 1.60) | 0.007 | 1.40(0.99, 1.98) | 0.059 | 1.25(1.07, 1.46) | 0.006 |
| HbA1c | 1.03(0.78, 1.36) | 0.836 | 1.03(0.78, 1.35) | 0.857 | 1.12(0.67, 1.88) | 0.666 | 1.02(0.85, 1.22) | 0.807 |
| **Multivariable Mendelian randomization** | | |  |  |  |  |  |  |
| IR | 1.24(1.02, 1.50) | 0.031 | 1.27(1.04, 1.54) | 0.018 | 1.62(1.23, 2.14) | 0.001 | 1.31(1.12, 1.52) | 0.001 |
| HbA1c | 1.08(0.82, 1.43) | 0.564 | 1.22(0.93, 1.60) | 0.148 | 1.10(0.84, 1.43) | 0.494 | 1.07(0.87, 1.33) | 0.515 |

In univariable MR, for HbA1c, rs579459 was removed because it was associated with heart failure at genome-wide significance level. *MR-PRESSO detected two significant outliers (rs132985 and rs966544) for IR and two significant outliers (rs10774625 and rs1558902) for HbA1c. In multivariable MR, rs2943645 cannot be extracted from the summary data of HbA1c, and rs579459 was also removed. After LD clumping at a threshold of r^2^ < 0.001 (clumping window: 10000 kB), leaving 72 independent SNPs as instrumental variables in the analysis. We removed four outliers found in univariable MR analysis as the result of MR-PRESSO.

**Table S14.** Heterogeneity and horizontal pleiotropy text of the associations between IR, HbA1c and heart failure in univariable and multivariate MR Analyses

|  | **IVW** | | | **MR-Egger** | | | | |
| --- | --- | --- | --- | --- | --- | --- | --- | --- |
|  | **Cochran’s Q** | | ***P-value*** | **Cochran’s Q** | ***P-value*** | **Intercept** | **SE** | ***P-value* for**  **intercept** |
| **Univariable Mendelian randomization** | |  |  |  |  |  |  |  |
| IR | 118.70 | | 3.97×10^-7^ | 116.13 | 5.47×10^-7^ | -0.004 | 0.004 | 0.293 |
| HbA1c | 93.34 | | 6.51×10^-7^ | 92.89 | 4.29×10^-8^ | -0.002 | 0.005 | 0.701 |
| **Multivariable Mendelian randomization** | | | | | |  |  |  |
| IR and HbA1c | 189.05 | | 6.85×10^-13^ | 172.16 | 8.74×10^-11^ | -0.007 | 0.003 | 0.009 |

IR, insulin resistance; HbA1c, hemoglobin A1c; IVW, inverse-variance weighted; LV, left ventricular.

**Figure S1.** Scatterplots of the causal estimates of glycemic traits and left ventricular end-diastolic volume.

| 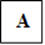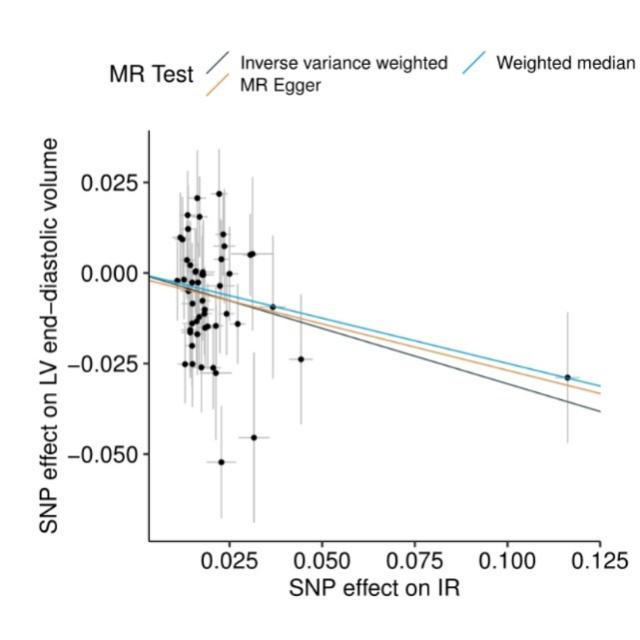 | 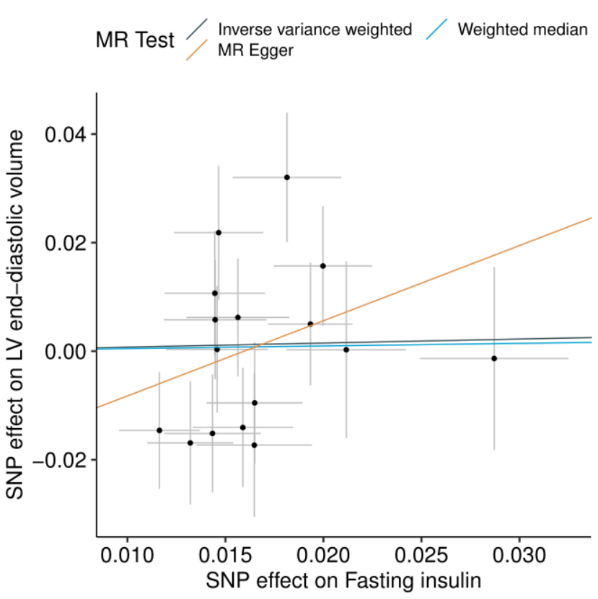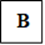 |
| --- | --- |
| 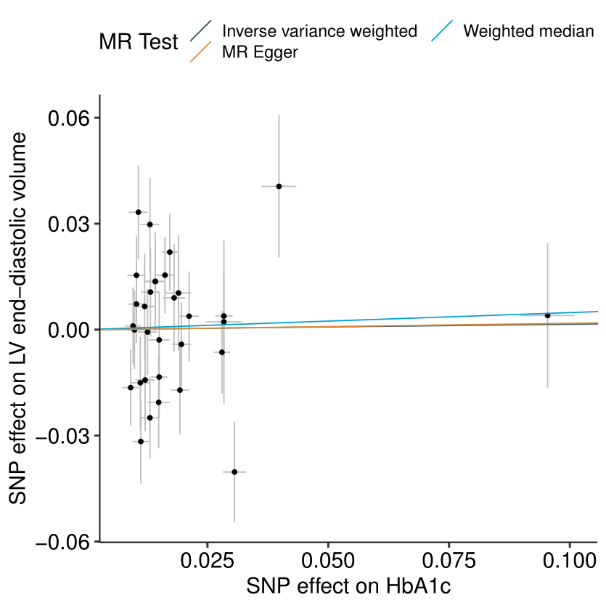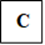 | 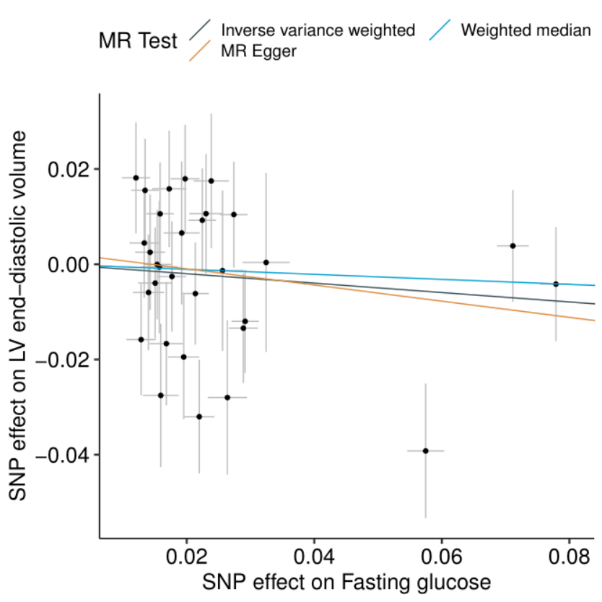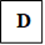 |

Scatterplots of SNP effects on IR (A), fasting insulin (B), HbA1c (C), fasting glucose (D) versus their effects on LV end-diastolic volume, with the slope of each line corresponding to the estimated MR effect of inverse variance-weighted, Weighted median, and MR-Egger, respectively. Error bars indicate 95% CIs. SNPs, single nucleotide polymorphisms; LV, left ventricular; IR, insulin resistance; HbA1c, hemoglobin A1c.

**Figure S2.** Scatterplots of the causal estimates of glycemic traits and left ventricular end-systolic volume.

| 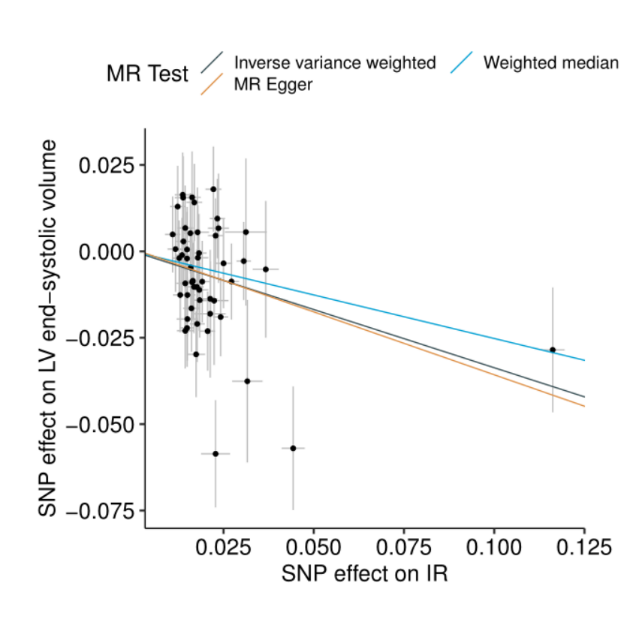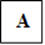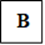 | 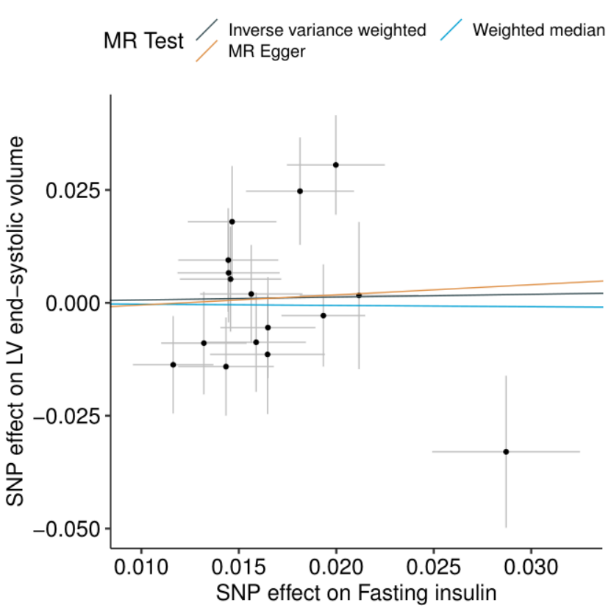 |
| --- | --- |
| 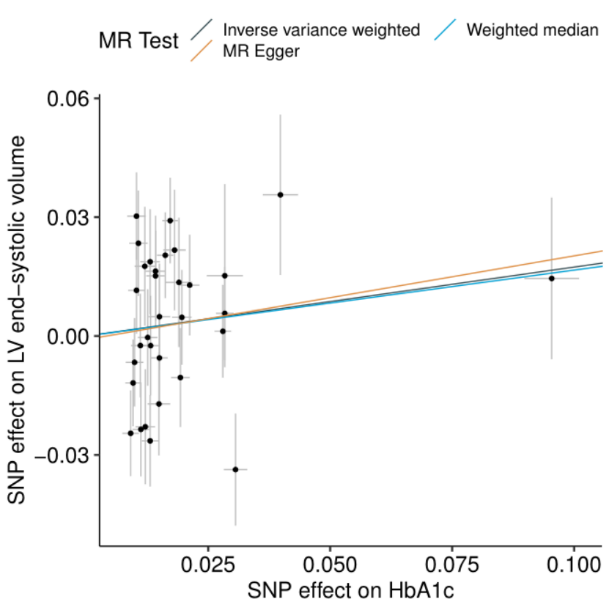 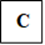 | 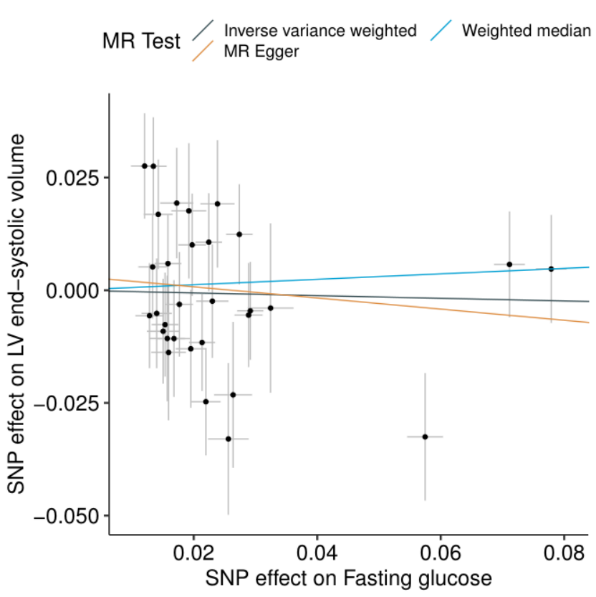 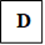 |

Scatterplots of SNP effects on IR (A), fasting insulin (B), HbA1c (C), fasting glucose (D) versus their effects on LV end-systolic volume, with the slope of each line corresponding to the estimated MR effect of inverse variance-weighted, Weighted median, and MR-Egger, respectively. SNPs, single nucleotide polymorphisms; LV, left ventricular; IR, insulin resistance; HbA1c, hemoglobin A1c.

**Figure S3.** Scatterplots of the causal estimates of glycemic traits and left ventricular ejection fraction.

| 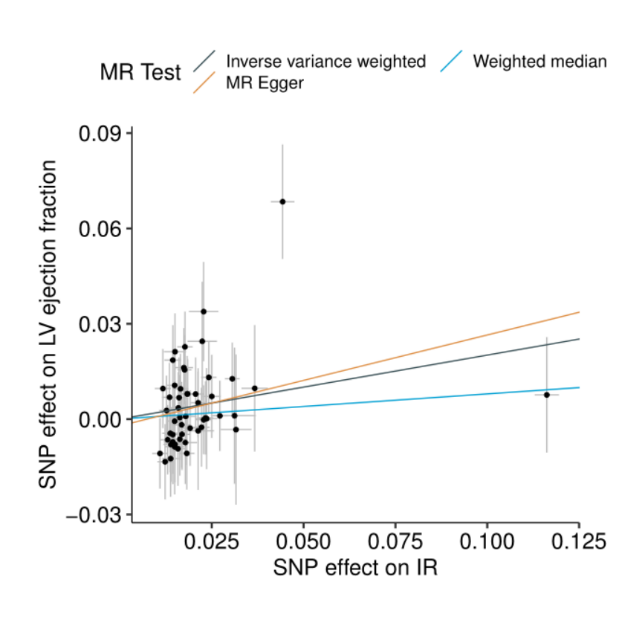 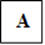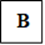 | 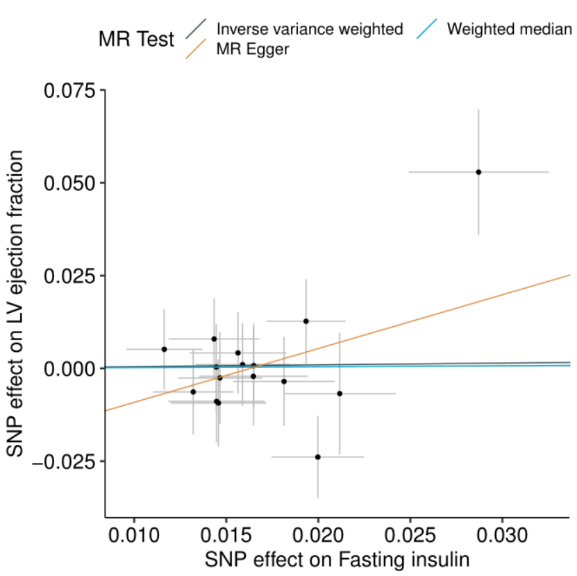 |
| --- | --- |
| 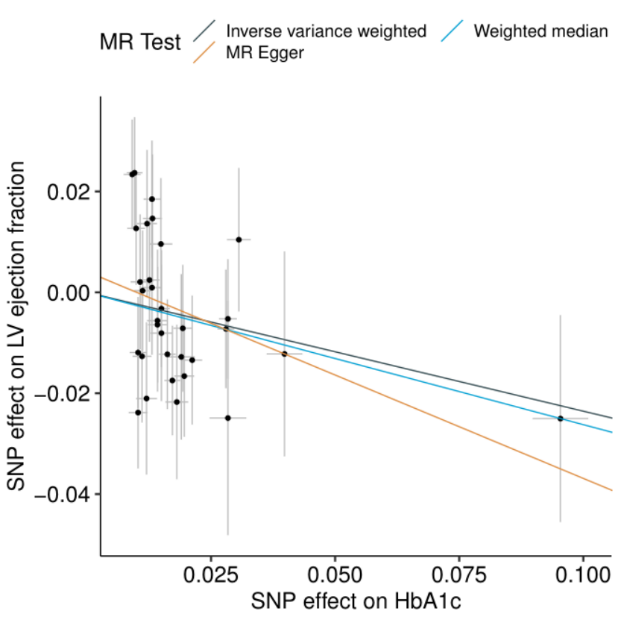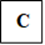 | 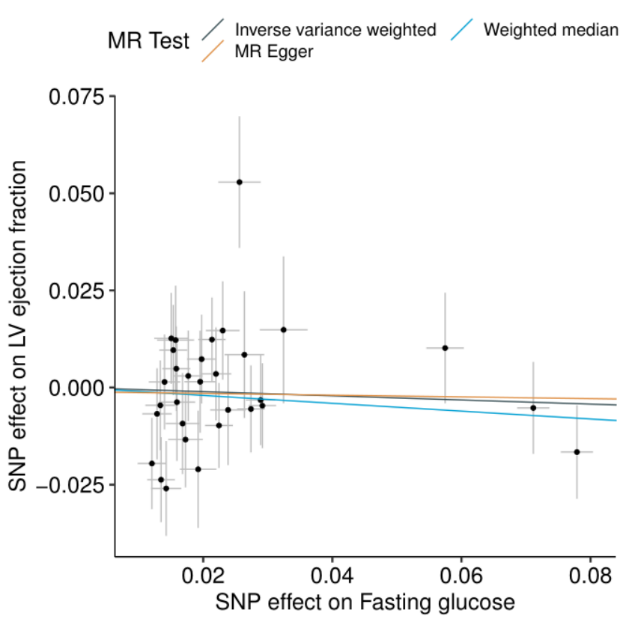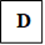 |

Scatterplots of SNP effects on IR (A), fasting insulin (B), HbA1c (C), fasting glucose (D) versus their effects on LV ejection fraction, with the slope of each line corresponding to the estimated MR effect of inverse variance-weighted, Weighted median, and MR-Egger, respectively. SNPs, single nucleotide polymorphisms; LV, left ventricular; IR, insulin resistance; HbA1c, hemoglobin A1c.

**Figure S4.** Scatterplots of the causal estimates of glycemic traits and left ventricular mass.

| 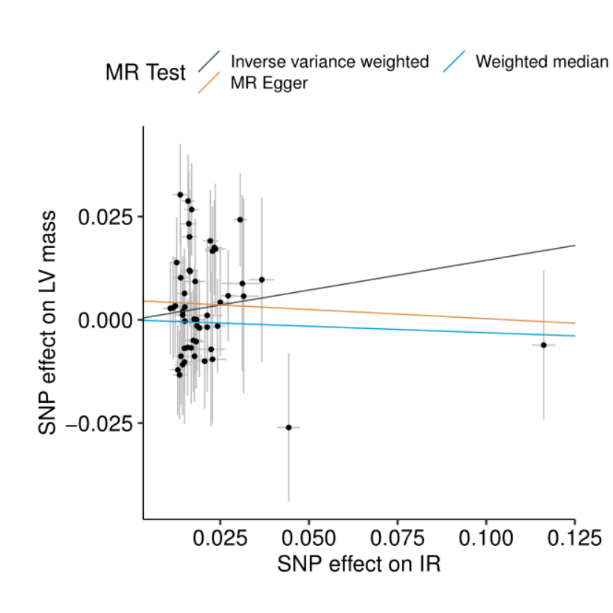 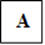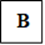 | 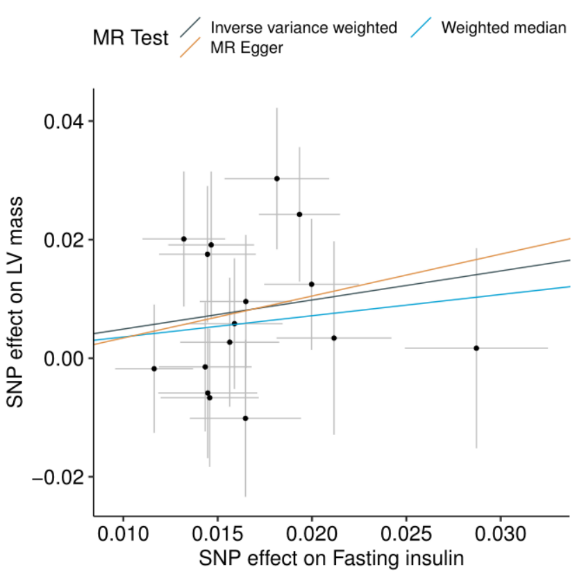 |
| --- | --- |
| 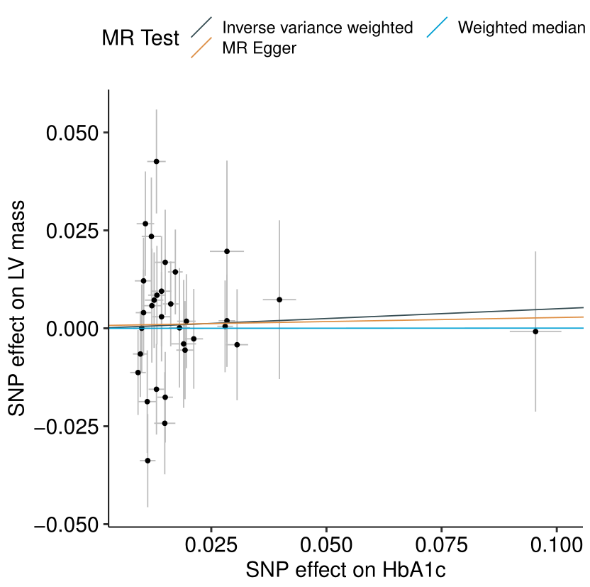 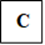 | 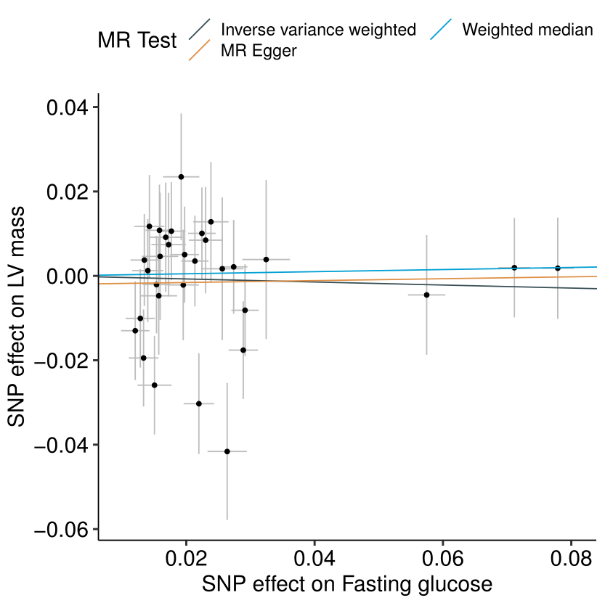 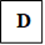 |

Scatterplots of SNP effects on IR (A), fasting insulin (B), HbA1c (C), fasting glucose (D) versus their effects on LV mass, with the slope of each line corresponding to the estimated MR effect of inverse variance-weighted, Weighted median, and MR-Egger, respectively. SNPs, single nucleotide polymorphisms; LV, left ventricular; IR, insulin resistance; HbA1c, hemoglobin A1c.

**Figure S5.** Scatterplots of the causal estimates of glycemic traits and left ventricular mass to end-diastolic volume ratio.

| 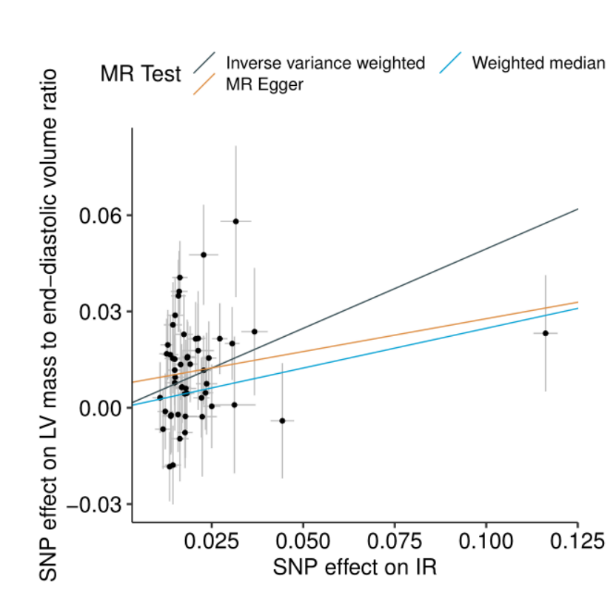 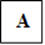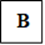 | 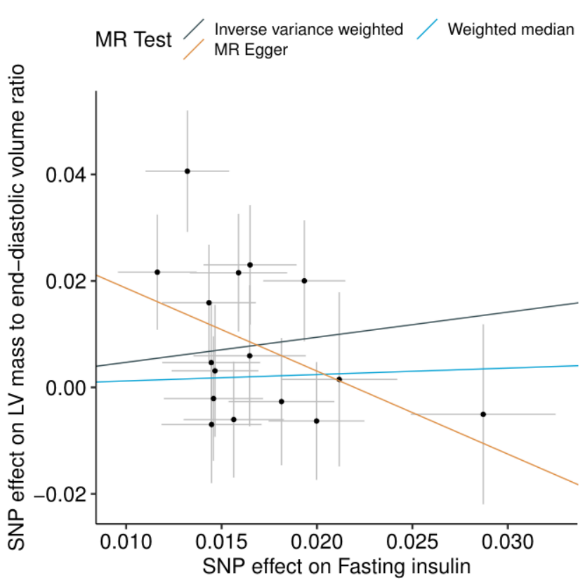 |
| --- | --- |
| 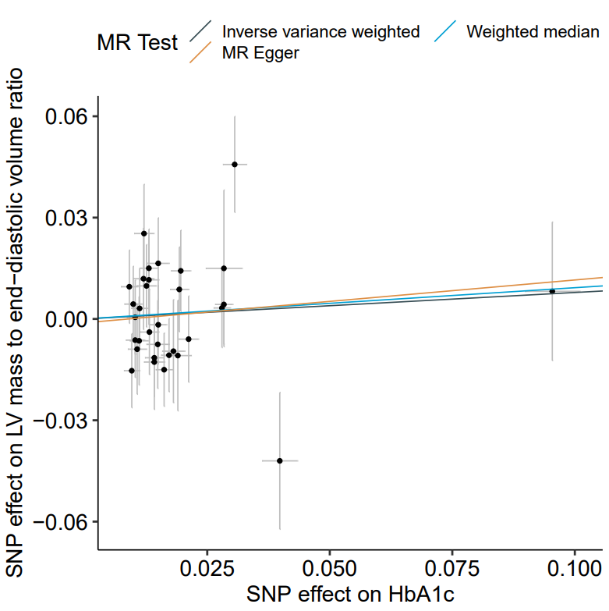 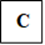 | 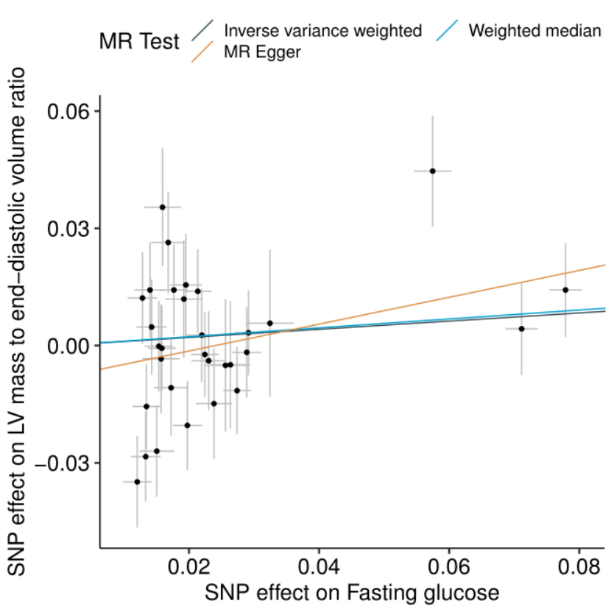 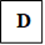 |

Scatterplots of SNP effects on IR (A), fasting insulin (B), HbA1c (C), fasting glucose (D) versus their effects on LV mass to end-diastolic volume ratio, with the slope of each line corresponding to the estimated MR effect of inverse variance-weighted, Weighted median, and MR-Egger, respectively. SNPs, single nucleotide polymorphisms; LV, left ventricular; IR, insulin resistance; HbA1c, hemoglobin A1c.


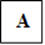
**Figure S6.** Leave-one-out analyses of the association between glycemic traits and left ventricular end-diastolic volume.

| 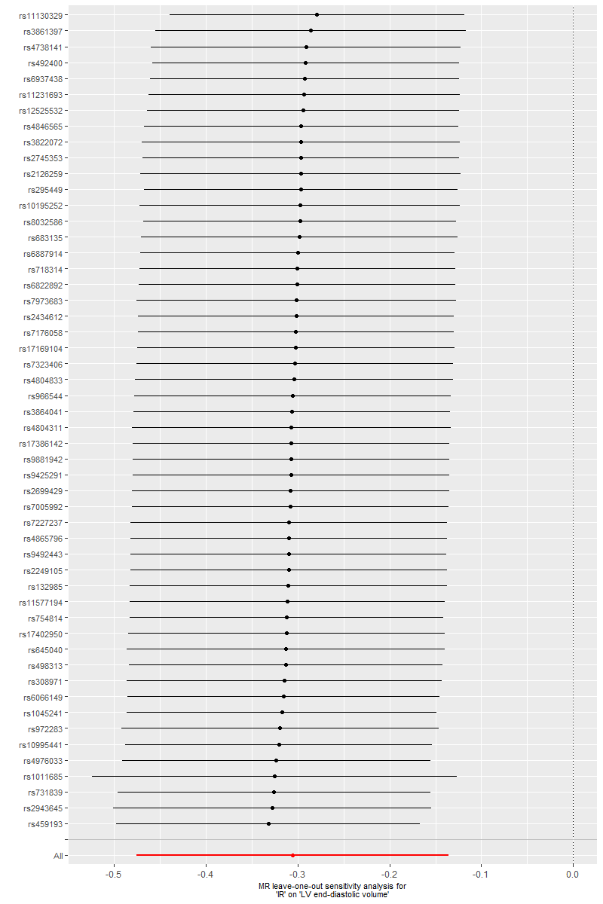 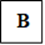 | 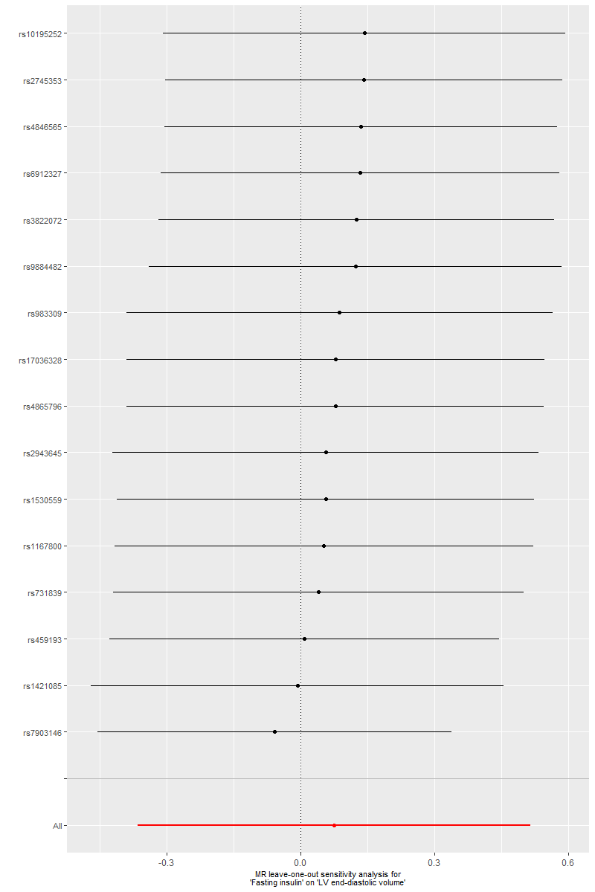 |
| --- | --- |
| 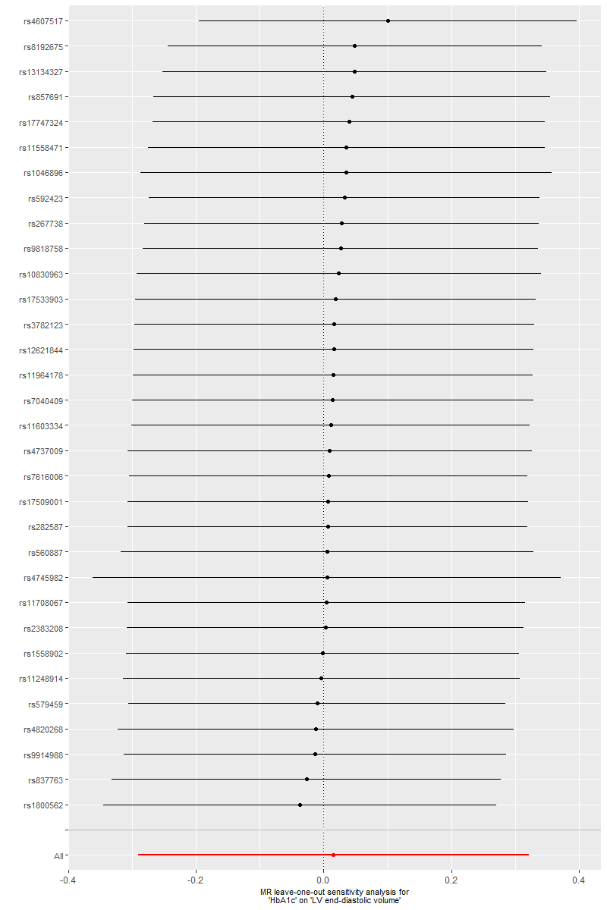 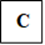 | 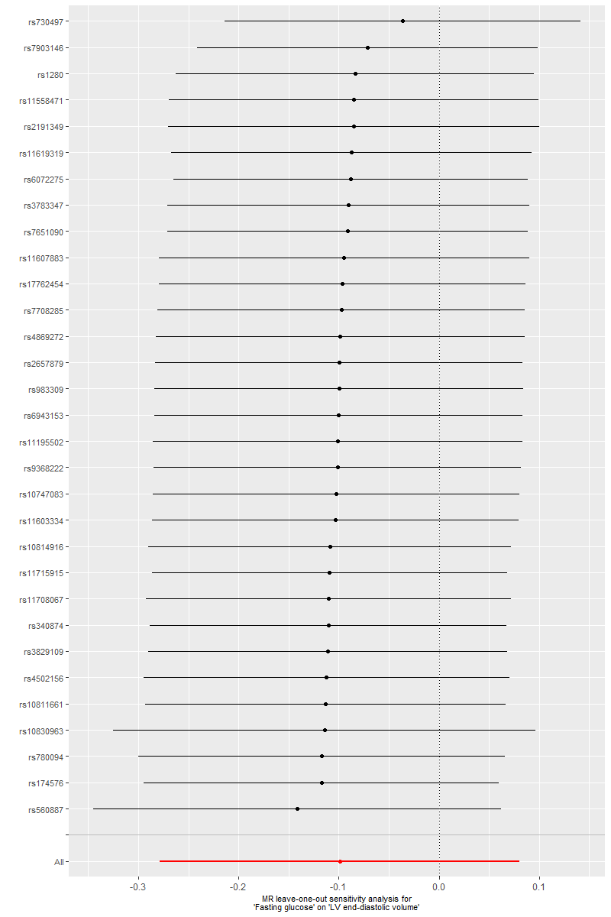 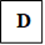 |

Leave-one-out analyses of the association between IR (A), fasting insulin (B), HbA1c (C), fasting glucose (D) and LV end-diastolic volume. LV, left ventricular; IR, insulin resistance; HbA1c, hemoglobin A1c.

**Figure S7.** Leave-one-out analyses of the association between glycemic traits and left ventricular end-systolic volume.

| 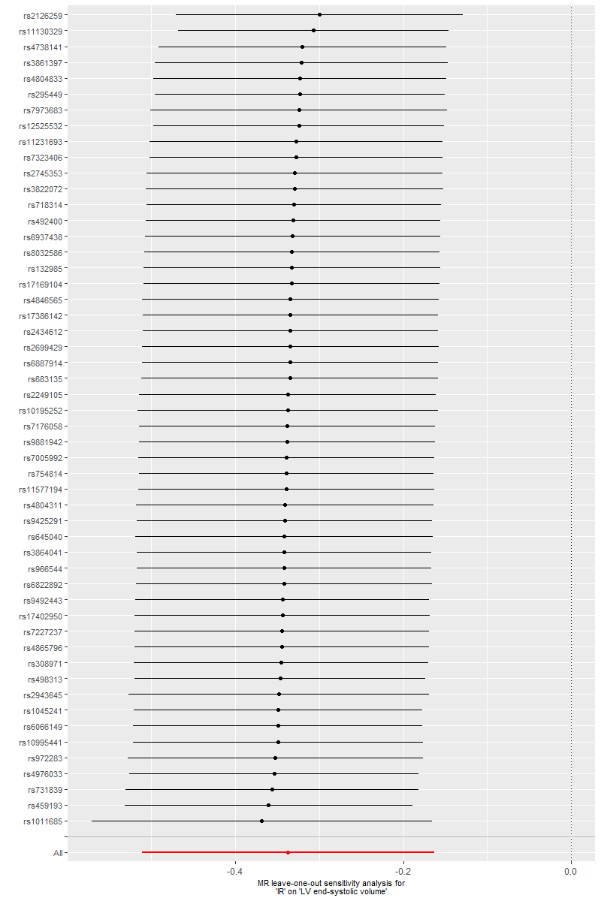 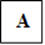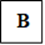 | 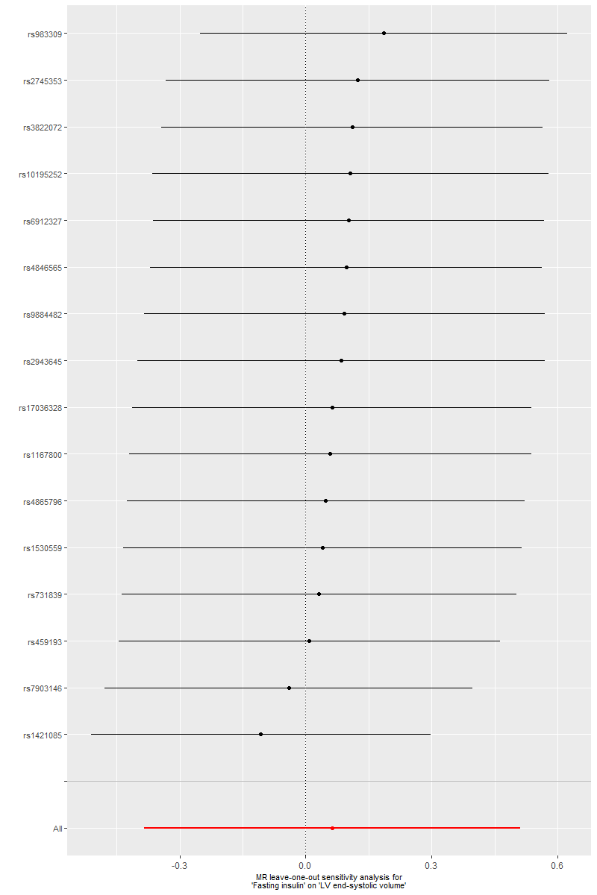 |
| --- | --- |
| 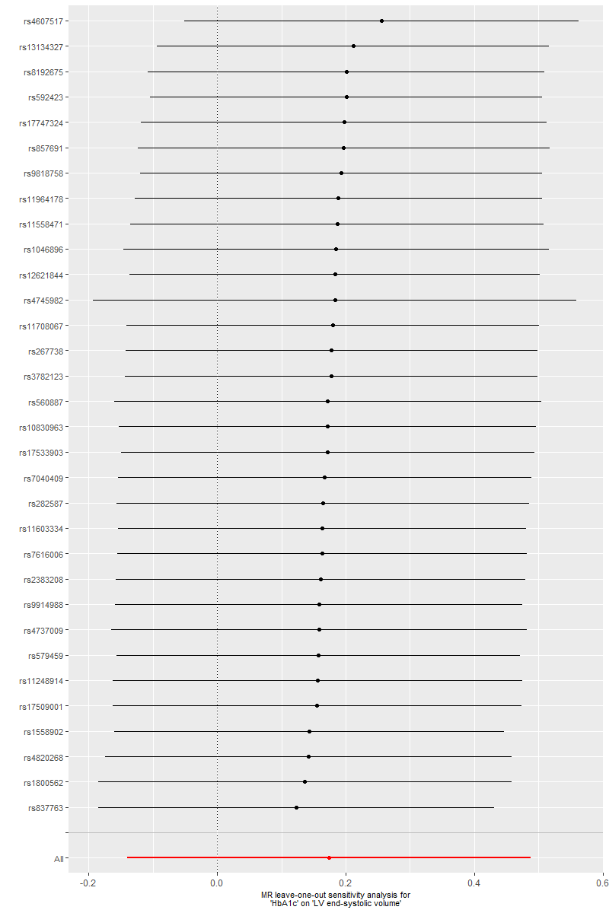 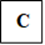 | 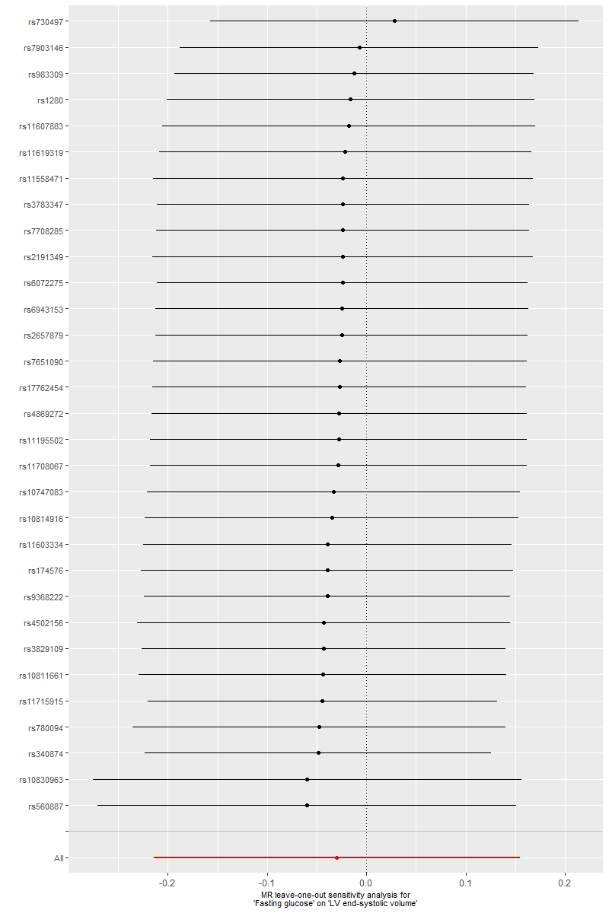 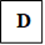 |

Leave-one-out analyses of the association between IR (A), fasting insulin (B), HbA1c (C), fasting glucose (D) and LV end-systolic volume. LV, left ventricular; IR, insulin resistance; HbA1c, hemoglobin A1c.

**Figure S8.** Leave-one-out analyses of the association between glycemic traits and left ventricular ejection fraction.

| 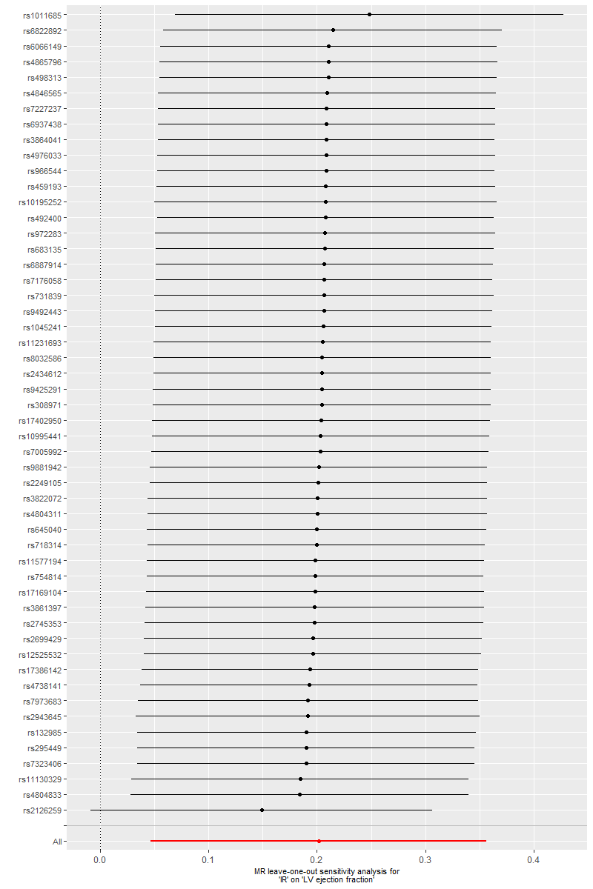 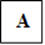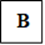 | 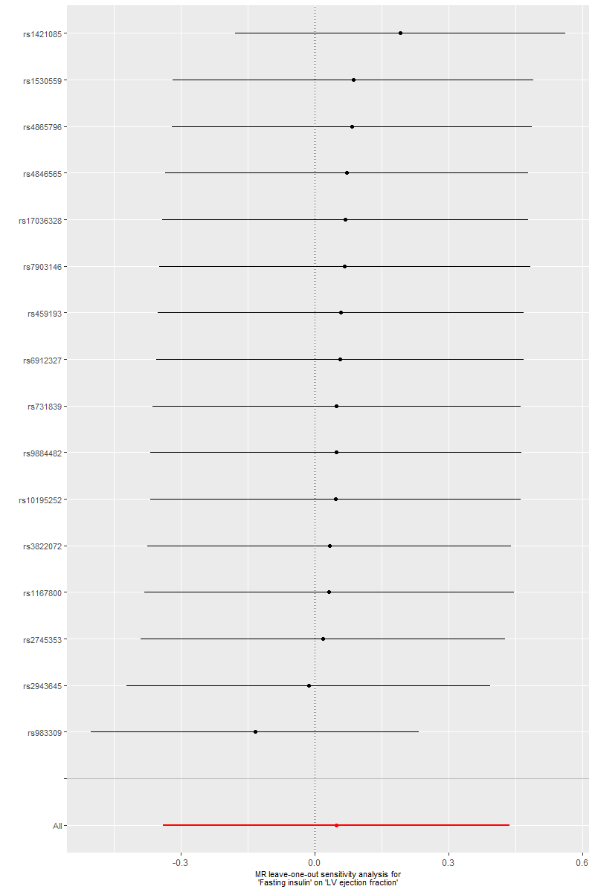 |
| --- | --- |
| 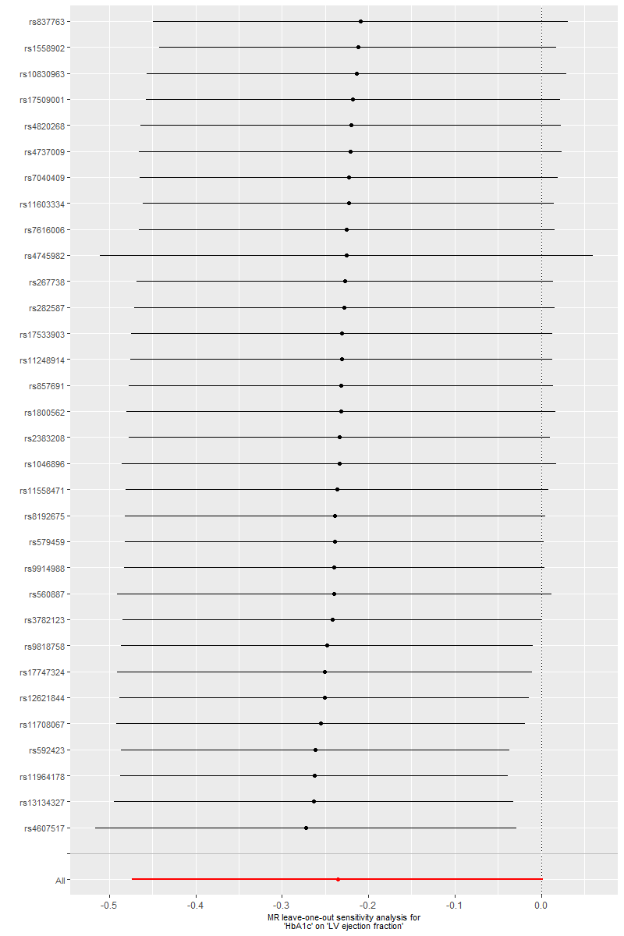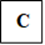 | 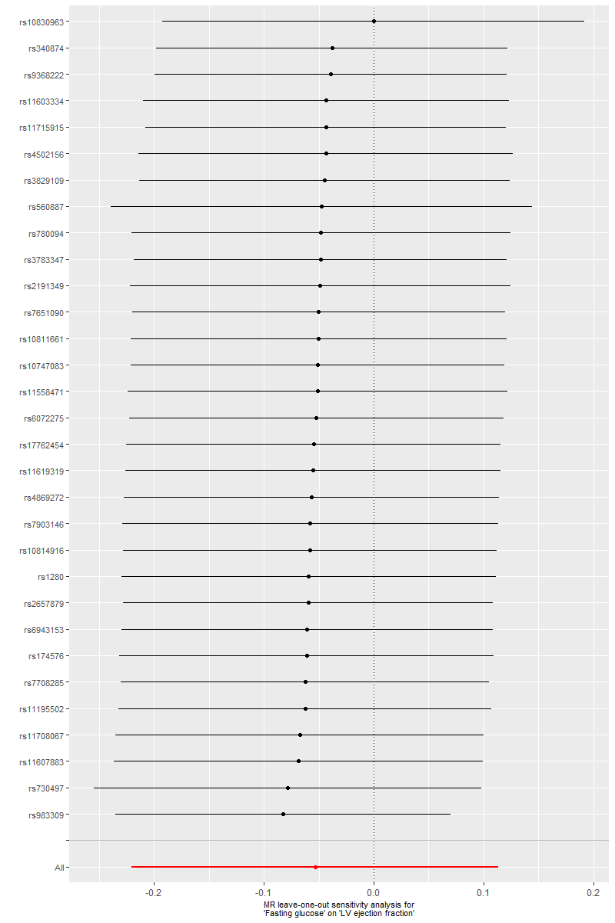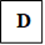 |

Leave-one-out analyses of the association between IR (A), fasting insulin (B), HbA1c (C), fasting glucose (D) and LV ejection fraction. LV, left ventricular; IR, insulin resistance; HbA1c, hemoglobin A1c.

**Figure S9.** Leave-one-out analyses of the association between glycemic traits and left ventricular mass.

| 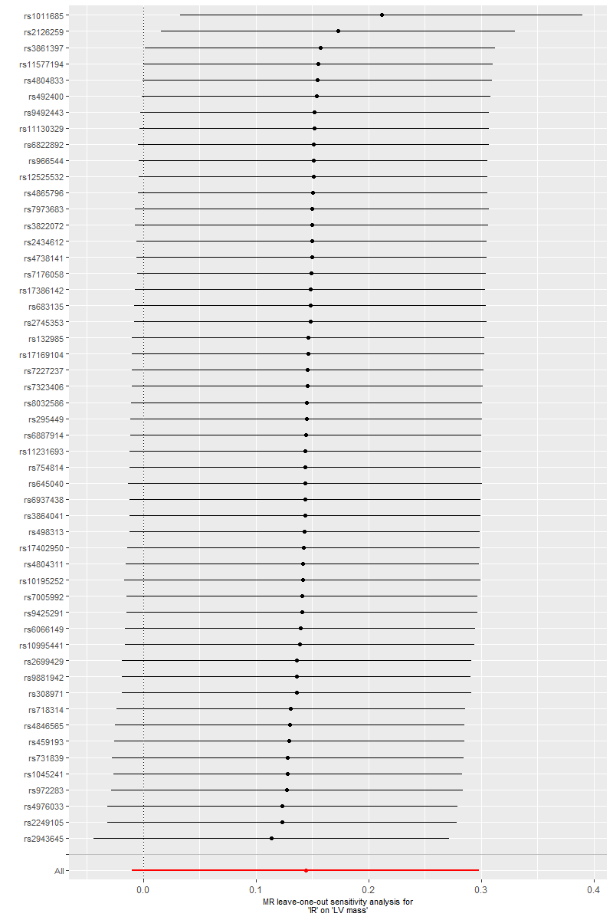 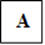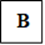 | 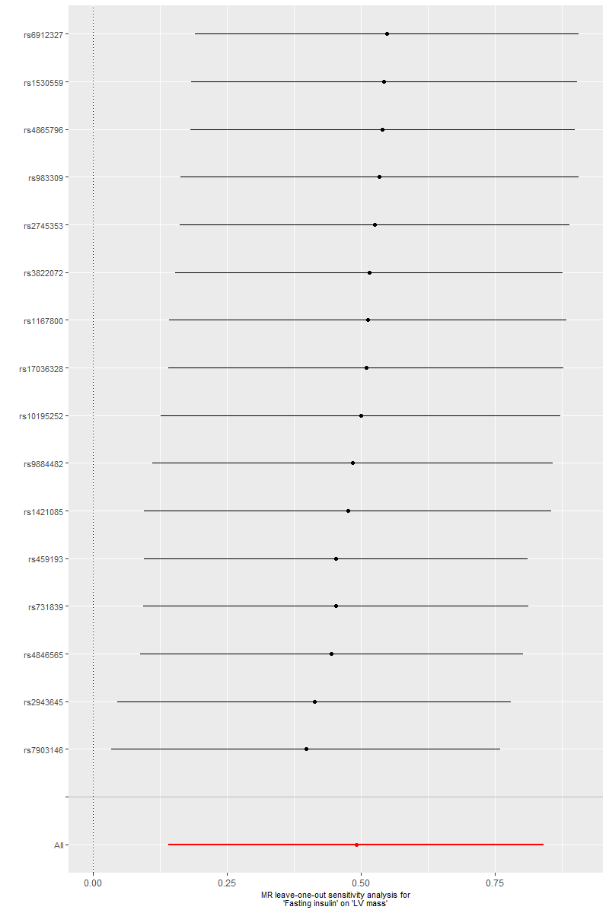 |
| --- | --- |
| 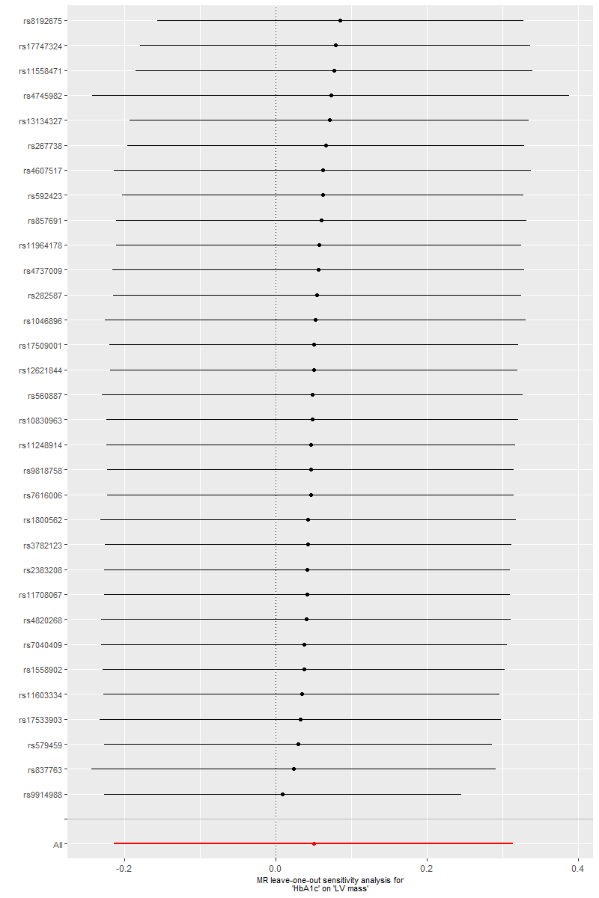 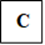 | 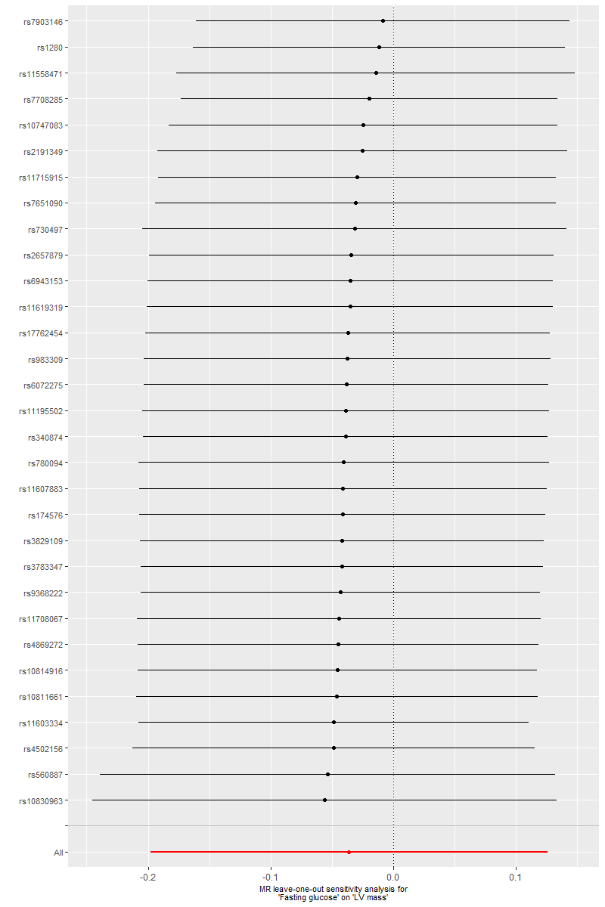 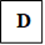 |

Leave-one-out analyses of the association between IR (A), fasting insulin (B), HbA1c (C), fasting glucose (D) and LV mass. LV, left ventricular; IR, insulin resistance; HbA1c, hemoglobin A1c.**Figure S10.** Leave-one-out analyses of the association between glycemic traits and left ventricular mass to end-diastolic volume ratio.

| 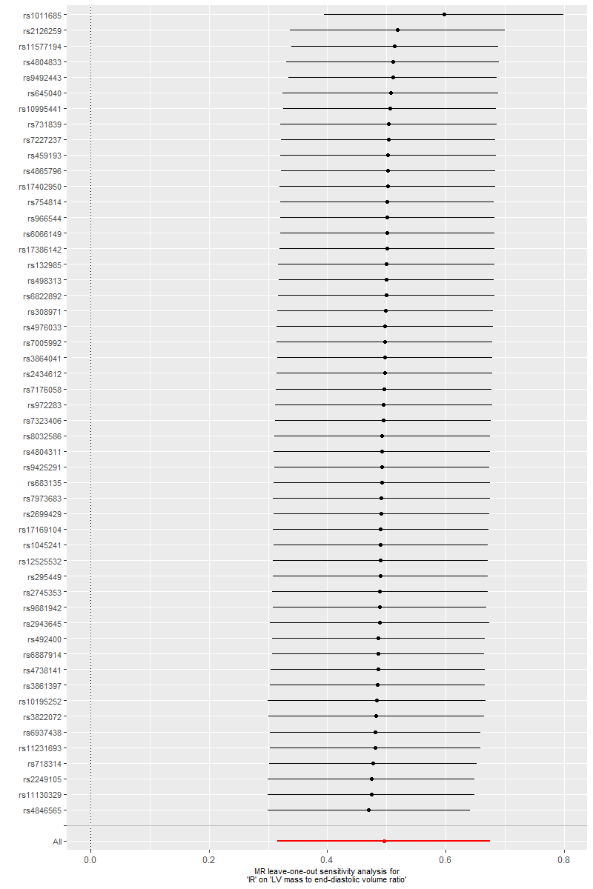 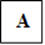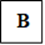 | 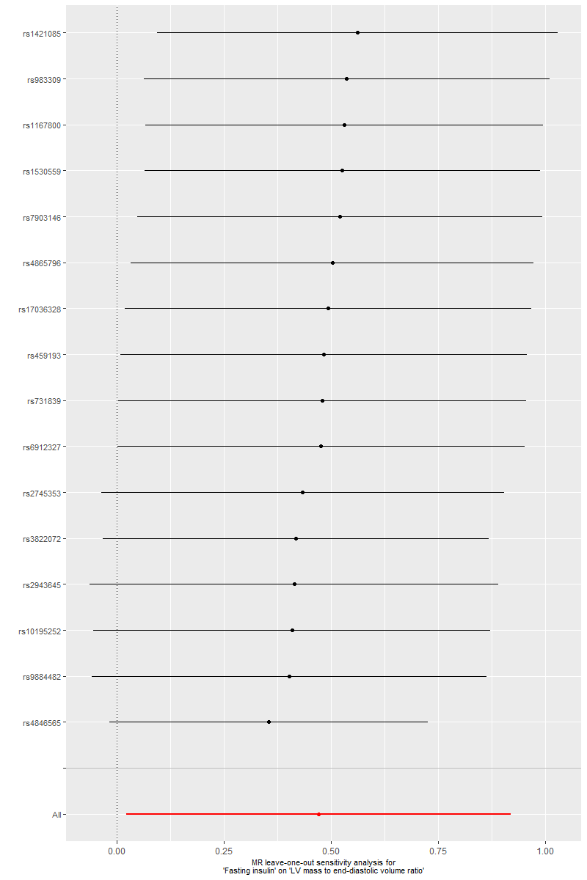 |
| --- | --- |
| 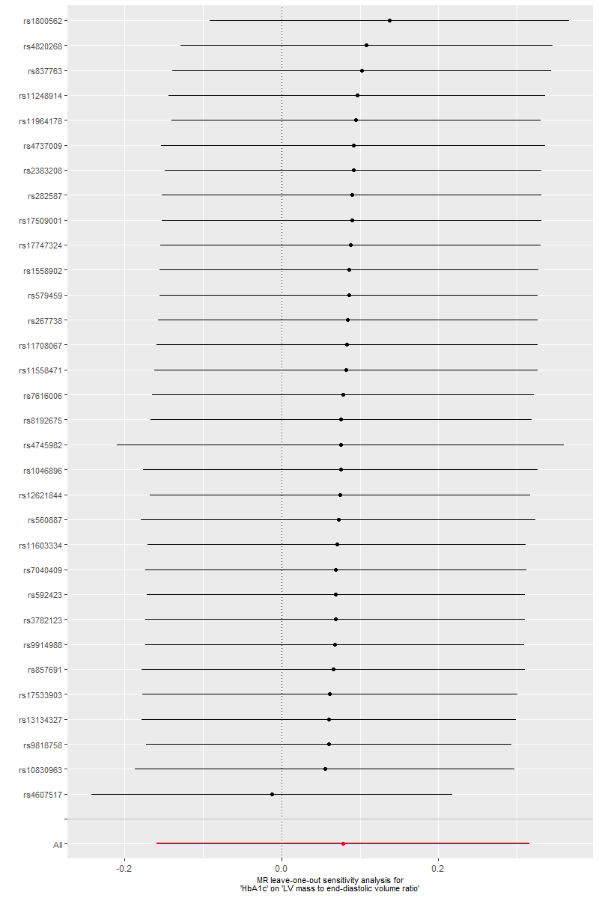 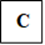 | 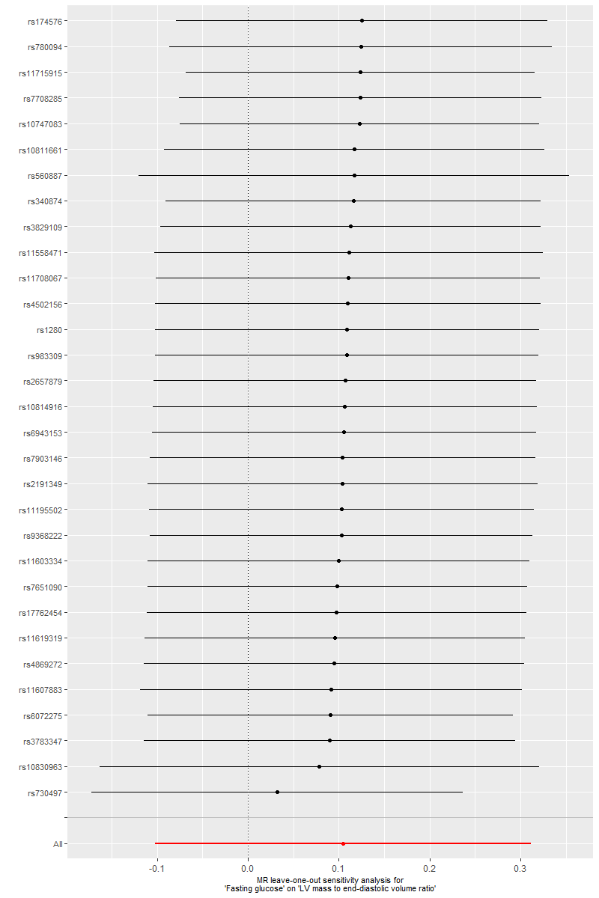 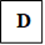 |

Leave-one-out analyses of the association between IR (A), fasting insulin (B), HbA1c (C), fasting glucose (D) and LV mass to end-diastolic volume ratio. LV, left ventricular; IR, insulin resistance; HbA1c, hemoglobin A1c.

**Figure S11.** Scatterplots of the bidirectional association of IR and HbA1c.

| 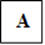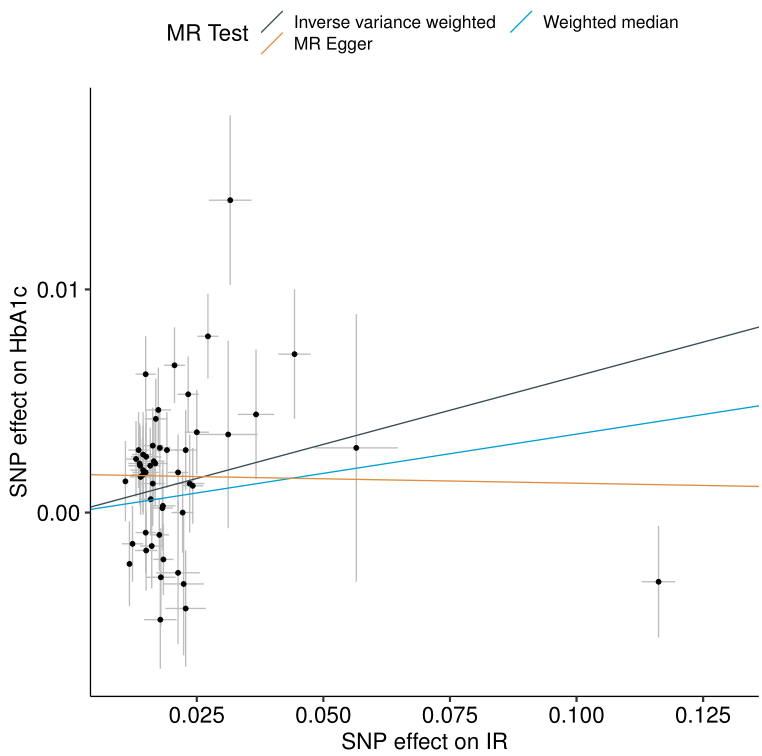 |
| --- |
| 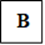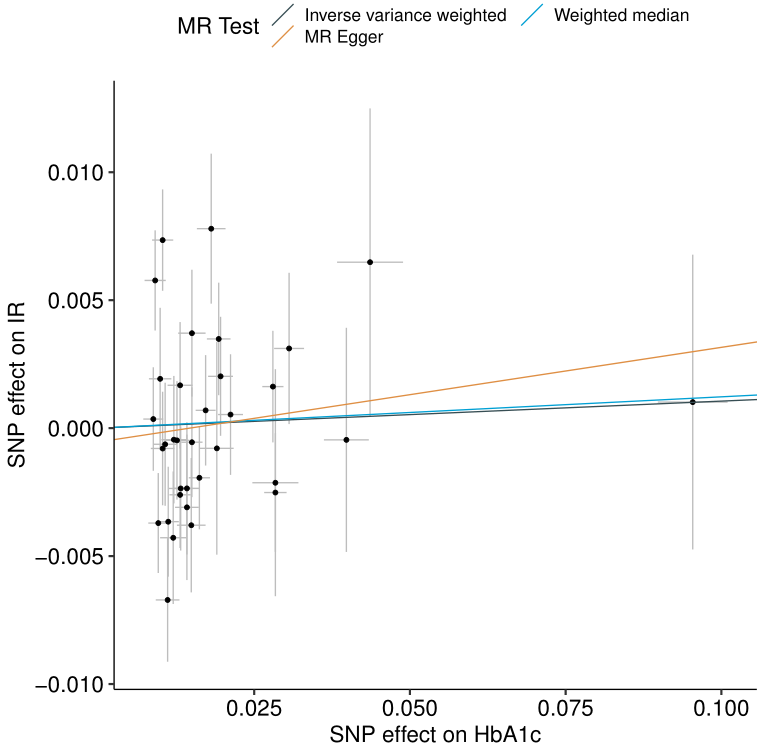 |

(A). Scatterplots of SNP effects on IR versus their effects on HbA1c, (B). Scatterplots of SNP effects on HbA1c versus their effects on IR. The slope of each line corresponds to the estimated MR effect of inverse variance-weighted, weighted median, and MR-Egger, respectively. Error bars indicate 95% CIs. SNPs, single nucleotide polymorphisms; IR, insulin resistance; HbA1c, hemoglobin A1c.

**Figure S12.** Leave-one-out analyses of the bidirectional association of IR and HbA1c.

| 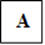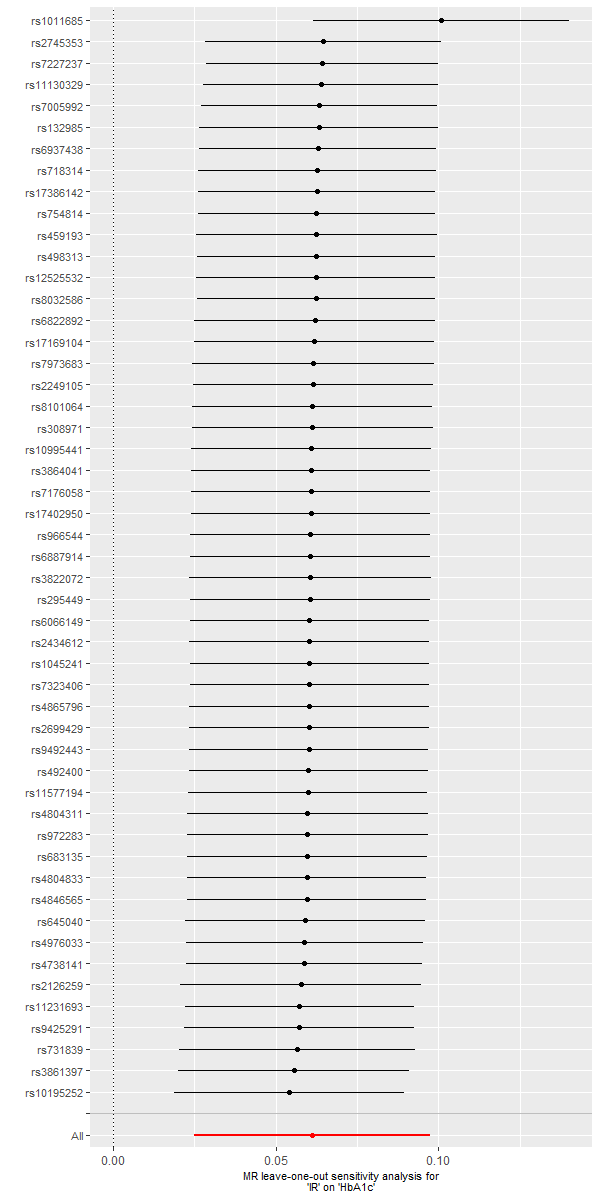 | 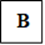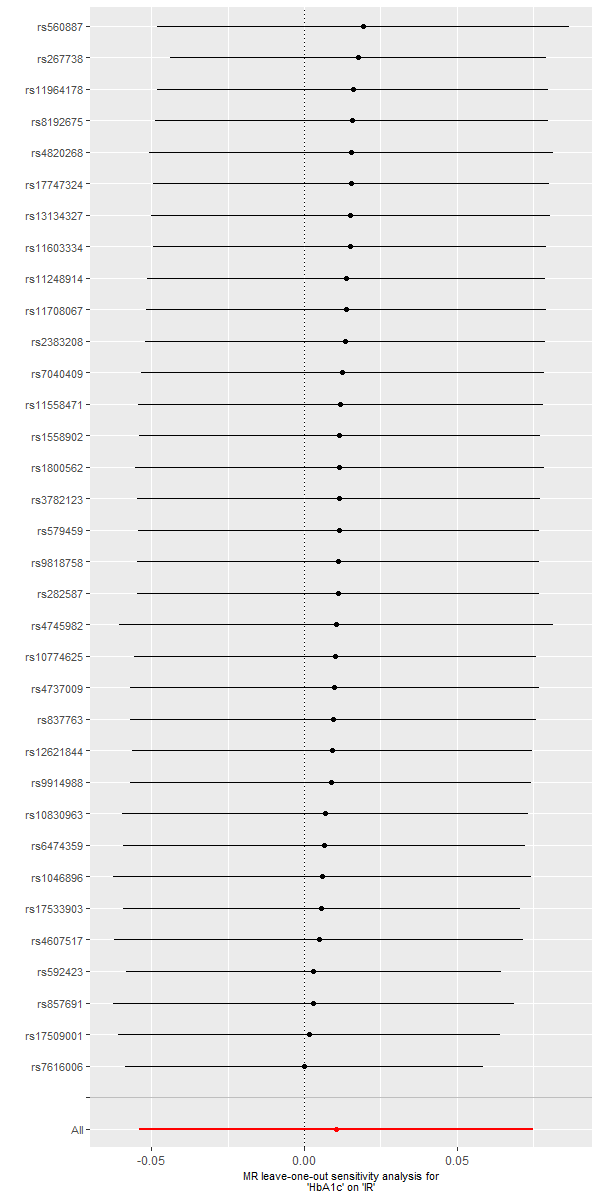 |
| --- | --- |

(A). Leave-one-out analyses of the association between IR and HbA1c, (B) Leave-one-out analyses of the association between HbA1c and IR. IR, insulin resistance; HbA1c, hemoglobin A1c.

**Figure S13.** A brief schematic diagram of possible assumption of the association of IR and HbA1c with cardiac morphology


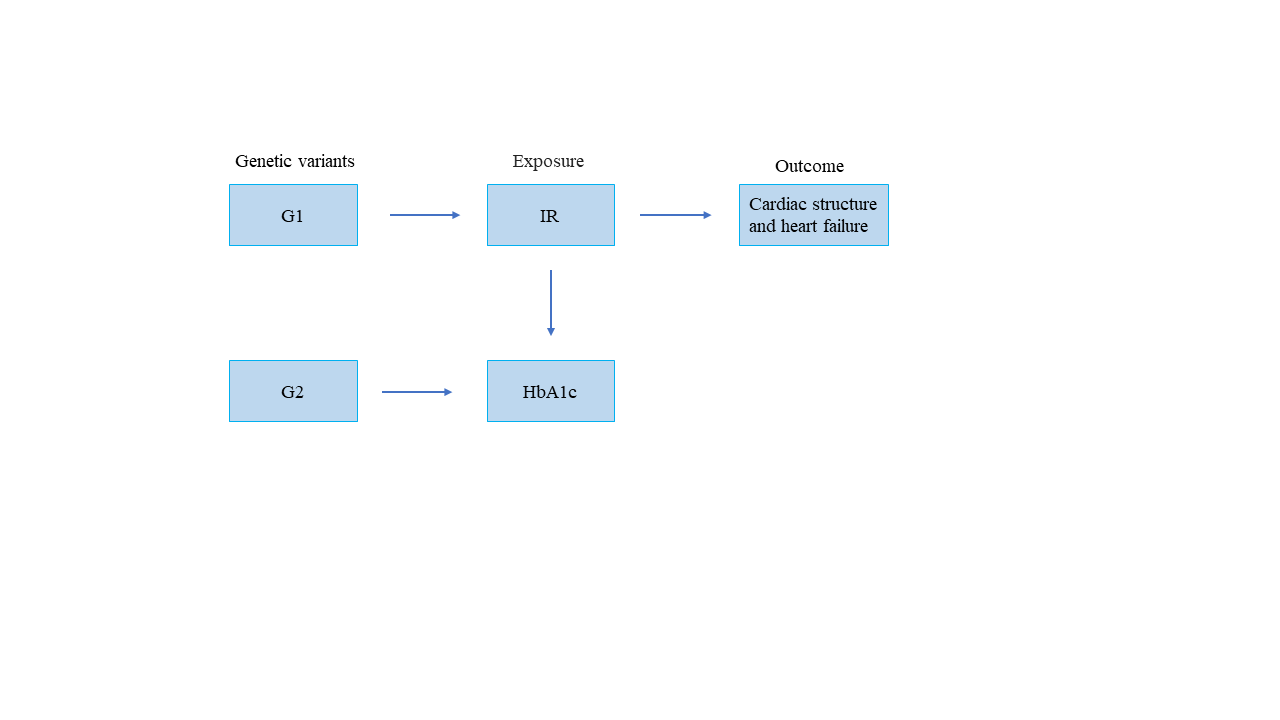


According to the univariable MR and multivariate MR analyses, our results suggested that genetic liability to IR rather than HbA1c is independently associated with adverse changes in LV parameters and heart failure risk. In bidirectional MR analyses, genetic liability to IR was significantly associated with higher HbA1c, whereas genetic liability to HbA1c did not result in significant alterations in IR. G, genetic variants; IR, insulin resistance.
